# Sequestration of Intestinal Acidic Toxins by Cationic Resin Attenuates Pancreatic Cancer Progression through Promoting Autophagic Flux for YAP Degradation

**DOI:** 10.3390/cancers14061407

**Published:** 2022-03-10

**Authors:** Guangfu Zhao, Tianci Zhang, Wei Liu, Mouad Edderkaoui, Richard Hu, Jun Li, Stephen J. Pandol, Xiangsheng Fu, Yuan-Ping Han

**Affiliations:** 1 The Center for Growth, Metabolism and Aging, College of Life Sciences, Sichuan University, Chengdu 610017, China; zgf515005117@outlook.com (G.Z.); 2017222040084@stu.scu.edu.cn (T.Z.); 2020222040007@stu.scu.edu.cn (W.L.); 2 Cedars-Sinai Medical Center, Los Angeles, CA 90001, USA; mouad.edderkaoui@cshs.org (M.E.); stephen.pandol@cshs.org (S.J.P.); 3 Olive View-UCLA Medical Center, Los Angeles, CA 90001, USA; richardhu@mednet.ucla.edu; 4 Department of Gastroenterology, Clinical Medical College and the First Affiliated Hospital of Chengdu Medical College, Chengdu 610083, China; 84183967@foxmail.com

**Keywords:** pancreatic cancer, yes-associated protein, bile acid, endotoxin, autophagy, lysosome, cystatin A

## Abstract

**Simple Summary:**

Annually, more than 450,000 people are diagnosed with pancreatic cancer worldwide with over 430,000 mortalities. Pancreatic ductal carcinoma (PDAC) accounts for around 80% of pancreatic cancer cases with an extremely high mortality rate. Emerging research has demonstrated that gut dysbiosis is closely associated with pancreatic cancer, while the underlying mechanisms remain elusive. In this study, we found that elevated levels of endotoxin (LPS) and bile acids were associated with malignant progression in Kras-driven pancreatic cancer mice. Importantly, oral administration of cationic resins to sequestrate intestinal endotoxins and bile acids efficiently attenuated tumor progression. Thus, sequestration of intestinal acidic toxins by oral administration of cationic resins may have potential as an intervention strategy for pancreatic malignancy.

**Abstract:**

Pancreatic cancer is driven by risk factors such as diabetes and chronic pancreatic injury, which are further associated with gut dysbiosis. Intestinal toxins such as bile acids and bacterial endotoxin (LPS), in excess and persistence, can provoke chronic inflammation and tumorigenesis. Of interest is that many intestinal toxins are negatively charged acidic components in essence, which prompted us to test whether oral administration of cationic resin can deplete intestinal toxins and ameliorate pancreatic cancer. Here, we found that increased plasma levels of endotoxin and bile acids in *Pdx1-Cre: LSL-Kras^G12D/+^* mice were associated with the transformation of the pancreatic ductal carcinoma (PDAC) state. Common bile-duct-ligation or LPS injection impeded autolysosomal flux, leading to Yap accumulation and malignant transformation. Conversely, oral administration of cholestyramine to sequestrate intestinal endotoxin and bile acids resumed autolysosomal flux for Yap degradation and attenuated metastatic incidence. Conversely, chloroquine treatment impaired autolysosomal flux and exacerbated malignance, showing jeopardization of p62/ Sqxtm1 turnover, leading to Yap accumulation, which is also consistent with overexpression of cystatin A (CSTA) in situ with pancreatic cancer cells and metastatic tumor. At cellular levels, chenodeoxycholic acid or LPS treatment activated the ligand–receptor-mediated AKT-mTOR pathway, resulting in autophagy-lysosomal stress for YAP accumulation and cellular dissemination. Thus, this work indicates a potential new strategy for intervention of pancreatic metastasis through sequestration of intestinal acidic toxins by oral administration of cationic resins.

## 1. Introduction

Pancreatic cancer, also known as pancreatic ductal adenocarcinoma (PDAC), has a dismal prognosis [1,2]. Most pancreatic cancers develop slowly from a premalignant lesion of pancreatic intraepithelial neoplasia (PanIN) which is associated with acquisition of the KRAS mutation. Moreover, *Ink4a/ARF* locus and *p53* inactivation in combination with *Kras* activation may further lead to metastasis and ultimate dissemination [3,4]. The benign form of PanIN can be maintained in dormancy for decades without dissemination, indicating that additional hits from environmental imprinting may participate in malignant transition [5,6,7,8].

The gut microbiome and commensal interaction with the host are essential for immunity, nutrition, and metabolic homeostasis, while gut dysbiosis can provoke chronic inflammation, insulin resistance, metabolic diseases, and various cancers [9,10,11]. Likewise, pancreatic cancer is tightly related to gut dysbiosis, showing downregulation of Firmicutes, while abundances of Actinobacteria and Proteobacteria, including *Escherichia Coli*, were upregulated [12,13,14]. Gut bacterial metabolites and toxins can enter portal and systemic circulation through paracellular diffusion or cotransport with chylomicrons [15,16,17]. Increased endotoxin levels were often found in the pancreatic tissue of PDAC patients, which was further related to the poor prognosis of PDAC under chemotherapy [18]. Another study found that plasma levels of endotoxin and IL-6 together with C-reactive protein (CRP) were increased in blood samples in line with duodenal bacterial up-localization in PDAC patients [19]. In animal models, administration of lipopolysaccharide (LPS) prolonged KRAS activation through the Ikk-NF-kappaB pathway, while deletion of the Ikk gene attenuated endotoxin-mediated malignancy [5]. Chronic pancreatitis and persistent inflammation are predisposing factors for the onset of PDAC [20]. Biliary obstruction and bile component influx, such as hydrophobic bile acids, were linked to digestive cancers such as PDAC, in part through oxidative stress, DNA damage, apoptosis, epigenetic regulation, and gut microbiome alteration [21]. High-level expression of FXR was found in the pancreatic tissues of PDAC with poor prognosis [22]. Furthermore, bacterial endotoxin from the gut could enter portal and systolic circulation with diet facilitated by chylomicrons; and plasma endotoxin and cytokines were increased with obesity [16]. Thus, sequestration of intestinal toxins may hold promise for prevention or even treatment of cancers such as PDAC.

The onset of PDAC in the elderly is related to the particular softness of pancreatic tissue, which may confer cellular dormancy for the genetically transformed cells [23,24]. Conversely, chronic pancreatic injury and pancreatic fibrosis, driven by the influx of gut metabolites and toxins, are critical for malignant transformation through stromal stiffness and immune tolerance [25,26,27,28]. YAP (yes-associated protein 1 or YAP1) is a transcription factor for cell proliferation and metastatic dissemination in a variety of cancers [29]. YAP is linked to the progression of pancreatic intraepithelial lesion (PanIN) to malignant PDAC, possibly via direct transformation of pancreatic epithelial cells or indirect influence on pancreatic stellate cells for fibrotic stiffness [30,31]. As a mechano-sensor and transcriptional activator in the sensing of substrate rigidity, YAP can be translocated into nuclei for transcriptional induction of tissue fibrosis and stiffness [32], which in turn may awaken dormant cancer cells to malignance. However, how gut metabolites can regulate YAP for PDAC development remains elusive. In this report, we found that elevated levels of plasma endotoxin and serum levels of bile acids are associated with impaired autolysosomal flux leading to Yap accumulation in metastatic malignancies. Oral administration of cationic resin can prevent metastasis in agreement with restoration of autolysosome flux for Yap degradation. In vitro, we found that LPS and hydrophobic bile acid could directly impede autolysosomal flux for Yap accumulation in PDAC cells. Thus, we propose a potential preventive application for pancreatic cancer through the administration of cationic resin to sequester intestinal acidic toxins.

## 2. Materials and Methods

### 2.1. Reagents, Plasmids, Primers, and Antibodies

Reagent and antibody information are presented in Supplementary File S2.

### 2.2. Animal Experiment

The animal practice, including treatments, followed the “Guide for Care and Use of Experimental Animals” by the National Research Council of the USA (Eighth Edition). The animal protocols were approved by the Institutional Animal Care and Use Committee (IACUC), the College of Life Sciences, Sichuan University. Mice were housed at 25 °C by IVC cages at the SPF level with free access to water and food. *Pdx1-Cre* mice were purchased from Biocytogen Beijing, and *LSL-Kras^G12D^* mice (KC mice) were from Sichuan University’s professor Xiao Zhixiong. Breeding was achieved through the crossing of *LSL-Kras^G12D^* mice with *Pdx1-Cre* mice. Genotyping of transgenic mice was performed by polymerase chain reaction (PCR) analysis. The primer sequences used for the genotyping of transgenic mice are presented in Supplementary File S2. For common bile duct ligation (BDL), mice were anesthetized with bromethol. Next, the peritoneal cavity was opened, and the common bile duct was double-ligated using 6-0 silk. Sham surgery was conducted in the peritoneal cavity, and two short 6-0 silks were placed under the common bile duct. Mice were sacrificed two weeks after surgery. For LPS treatment, the 12-week-old mice received an intraperitoneal injection of 4 mg/kg LPS or saline once a week for four consecutive weeks, followed by sacrifice a week after the last injection. For chloroquine (CQ) injection treatment, the 6-week-old mice were given an intraperitoneal injection of chloroquine at a dosage of 60 mg/kg or saline once a day for 10 weeks before sacrifice. In another treatment, chloroquine at 2 mg/mL in drinking water was given to the 6-week-old mice for 10 weeks. For cholestyramine treatment, the 6-week-old mice were fed with AIN93 chow containing 3% cholestyramine (*w*/*w*) for 14 consecutive weeks.

### 2.3. Histological Analysis

At the end of the experiment, mice were sacrificed and the pancreas, as well as other organs such as the liver and lungs, were collected. The tissues were immediately fixed with 4% paraformaldehyde (*w*/*v*), followed by embedding in paraffin and sectioning at 5 μm thickness. Hematoxylin and eosin (H&E) staining and Masson’s trichrome staining were performed according to the manufacturer’s instructions (Beyotime Biotechnology, Shanghai, China). Microscopic imaging was captured by NanoZoomer (Hamamatsu, Japan) and analyzed with Hamamatsu NDP view2 software. Characteristic analysis of acinar-to-ductal metaplasia (ADM) and grading of PanIN and PDAC were based on previously established criteria [33]. For quantification of ADM and PanIN lesions, the incidences of ADM and PanIN lesions in the whole mount were recorded. The results were scored by two trained laboratory technicians.

### 2.4. Immunohistochemistry

The ORIGENE kit was used to perform immunohistochemistry according to the manufacturer’s instructions (Beijing, China). Briefly, the dewaxed slides were incubated with specific antibodies according to recommended dilution overnight at 4 °C, followed by incubation with HRP-conjugated secondary antibodies for 40 min at room temperature. Slides were visualized using DAB staining and counterstained with hematoxylin. For quantification, three random fields were captured by NanoZoomer and analyzed with NDP view2 software.

### 2.5. Determining Blood Levels of Endotoxin and Total Bile Acids

The Limulus amebocyte extract kit (catalog no. CE80545, Biondo, Xiamen, China) was used to measure the plasma concentration of LPS. The plasma LPS concentration was measured by dilution in a sample processing buffer at a 1:10 ratio and heated for 10 min at 70 °C. The concentration of total bile acid in serum was measured by the total bile acid kit (catalog no. E003-2-1, Nanjing Jiancheng Bioengineering Institute, Nanjing, China) according to the manufacturer’s instructions. Briefly, serum samples and reagents were added to 96-well plates and then incubated at 37 °C, after which the optical density (OD) was measured at 490 nm using a multifunctional microplate reader (BioTek, Winooski, VT, USA).

### 2.6. Western Blotting Analysis

Briefly, cells in culture wells were lysed with RIPA buffer containing protease inhibitor cocktail. For tissues, a homogenization step was needed. After centrifugation, the protein concentration in supernatants were quantified by bicinchoninic acid (BCA) method and boiled with reducing agent. Samples were resolved by SDS–PAGE, followed by transfer to a PVDF membrane. Primary antibodies were incubated on the blotted PVDF membrane overnight at 4 °C, and then HRP-conjugated secondary antibodies were applied to the membrane for 1 h at room temperature. Finally, the blots were visualized with an ECL kit (Bio–Rad, Hercules, CA, USA). Western bands were quantified using Bio–Rad Image Lab software and normalized by beta-actin or gapdh.

### 2.7. Cell Lines and Transfection

UN-KPC-961 (*Kras^G12D^, Trp53^R172H^, Pdx1-Cre*) and UN-KC-961 (*Kras^G12D^, Pdx1-Cre*) cells were gifts from Professor Stephen J. Pandol at the Cedars-Sinai Medical Center [34]. BxPC-3 (*RAS, TP53^Y220C^*), PANC-1 (*KRAS^G12D^, TP53^R273H^*), Mia-PaCa-2 (*KRAS^G12C^, TP53^R248W^*), and HEK293FT cells were bought from ATCC (American Tissue Culture Collection, Beijing). Cells were cultivated at atmosphere with 5% CO_2_ at 37 °C by Dulbecco’s modified Eagle medium supplemented with 10% fetal bovine serum and 1% penicillin/streptomycin. BxPC-3 cells were maintained in RPMI-1640 medium supplemented with 10% fetal bovine serum and 1% penicillin/streptomycin. Lipofectamine 3000 was used to perform DNA transfection (Thermo Fisher, Waltham, MA, USA) according to the manufacturer’s instructions.

### 2.8. Generation of Lentivirus

Lentivirus was generated by co-transfection with plasmid pLKO.1 (for shRNA) or pLVX-Puro (for overexpression) together with packaging vectors pMDG.2 and psPAX2 on HEK293FT cells by transfection agent Lipo3000 (Thermo Fisher, USA). The sequence information for shRNA knockdown and overexpression is provided in Supplementary File S2.

### 2.9. Measurement of Autophagic Flux Using the Autophagy Tandem Sensor mRFP-GFP-LC3

Cells stably expressing LC3-tandem-mRFP-GFP were cultured in 24-well plates for 24 h and then treated with the indicated conditions as described in the text. Microscopic images were captured by a confocal microscope (Leica TCS SP5II, Wetzlar, Germany). The red and yellow puncta represent autophagosomes and autolysosomes, respectively, and red puncta indicate degradation of GFP-LC3 and strong autophagic flux.

### 2.10. Transwell Cell Migration Assay

The assays were performed using 8.0 µm pore-size Transwell inserts (Corning, New York, NY, USA). Briefly, 50,000 cells were suspended in 300 µL serum-free medium and seeded into the upper chambers, while 500 µL of complete medium was added to the lower chamber as a source of chemoattractant. Cells were cultured for 12–16 h at 37 °C, followed by fixing with cold 100% methanol and then stained with 0.1% crystal violet. The nonmigrating cells were gently removed with cotton swabs. Cell migration was determined by counting the stained cells under a light microscope in three randomly selected fields. The experiments were repeated three times.

### 2.11. Clone Formation Assay

An approximate count of 2000 cells was seeded into a 6-well plate. Cold 100% methanol and 0.1% crystal violet were used to fix and stain the wells after 7–10 days of culturing. Colonies larger than 0.5 mm in diameter were counted under the microscope. The experiments were repeated three times.

### 2.12. Cell Viability Assay

Cell viability was measured using Cell Counting Kit-8 according to the manufacturer’s instructions (Abclonal, Wuhan, China). Briefly, cells were seeded into 96-well plates at a density of 2000 cells per well and incubated overnight in 10% FBS medium with or without drugs. After 12 h of incubation at 37 °C with 5% CO_2_, 10µL of CCK-8 solution was added to each well and incubated for another 4 h at 37 °C. Then, the optical density was measured at 450 nm on a multifunctional microplate reader (BioTek Synergy H1, Winooski, VT, USA).

### 2.13. Statistical Analysis

For statistical analyses, GraphPad Prism software (Prism 6.0) and PASW Statistical 18.0 software (SPSS) were used. Groups of individual data are expressed as the means ± standard deviation (SD). Student’s *t*-test (two-tailed), Mann–Whitney test (two-tailed), or one-way ANOVA were used to determine the significance of the in vitro and in vivo data. Survival rates were described by a Kaplan–Meier curve and quantified by a log rank (Mantel–Cox) test. Statistical significance is represented as followed. *: *p* < 0.05, **: *p* < 0.01, ***: *p* < 0.001.

## 3. Results

### 3.1. Elevated Levels of Systemic Endotoxin and BILE Acids in Pdx-1-Kras Mice Are Associated with Metastatic Progression in Mice

**Figure 1:**
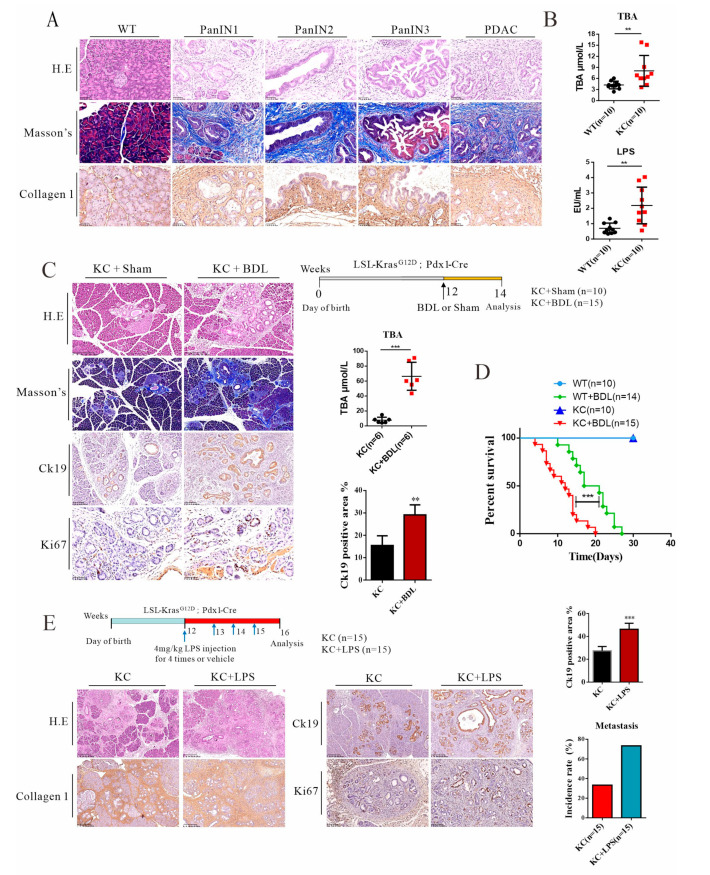
Elevated levels of systemic endotoxin and bile acids in Pdx-1-Kras mice are associated with metastatic progression in mice. (**A**) KC mice recapitulated the pathological characteristics of pancreatic cancer. Normal pancreatic tissue and different stages of pancreatic cancer—including PanIN1, PanIN2, PanIN3, and invasive PDAC—were illustrated by hematoxylin and eosin (H&E) staining and Masson’s trichrome staining, as well as immunohistochemical staining of type 1 collagen. Scale bars = 50 μm. (**B**) Serum total bile acid and plasma endotoxin levels. (**C**) KC mice were subjected to bile duct ligation (BDL) or a sham procedure, as indicated by a scheme, KC + Sham (*n* = 10) and KC + BDL (*n* = 15) respectively. The pancreatic tissues were stained with H&E and Masson’s trichrome. Scale bars = 250 μm. Representative images show pancreatic precursor lesions stained with Ck19 (scale bars = 100 μm) and Ki67 (scale bars = 50 μm) and quantitation of the percentage of CK19-positive duct-like structures. (**D**) Kaplan–Meier survival analysis of KC mice subjected to sham or BDL treatment. (**E**) A scheme depicts the protocol for KC mice that were given either a vehicle or LPS treatment. KC + vehicle (*n* = 15) and KC + LPS (*n* = 15). Pancreatic tissues were stained with H&E (scale bars = 250 μm), type 1 collagen (scale bars = 250 μm), Ck19 (scale bars = 250 μm), and Ki67 (scale bars = 100 μm). Quantitation of the percentage of CK19-positive area and metastasis incidence rate. Representative images of histological staining and IHC staining are shown. ** *p* < 0.01, *** *p* < 0.001.

Pdx1-Kras mice (KC) recapitulated pathophysiological features for the transition of PanIN to PDAC. Low-grade pancreatic intraepithelial neoplasia (PanIN1 and 2) was indicated (Figure 1A), featuring flat epithelial lesions consisting of tall columnar cells with nuclei located basally and abundant in supranuclear mucus. Similarly, high-grade pancreatic intraepithelial neoplasia in PanIN-3 and PDAC, consisting of atypical tubular and papillary glands, was also evident in the histological analysis. Importantly, conspicuous fibrosis and stromal stiffness, indicated by Masson’s trichrome staining and fibrotic type-I collagen deposition, were in line with the growth of PanIN and transition to PDAC, suggesting activation of pancreatic stellate cells and building up tissue stiffness for the malignant transition. Serum levels of bile acids and plasma levels of endotoxin were measured, and both had an approximately two-fold increment (Figure 1B), which is in agreement with clinically observed hyperbileacidemia and hyperendotoxemia in association with chronic pancreatitis and PDAC.

Biliary obstruction and its associated jaundice are often related to pancreatic cancer [35,36]. Here, we performed a surgical procedure for common bile duct ligation (BDL) in KC mice. As anticipated, bile duct ligation led to jaundice and increased influx of bile acids into plasma, leading to mortality as well (Figure 1C,D). Bile duct ligation provoked severe pancreatic lesions and capillary ductal growth, showing increased CK19-positive cells and Ki67-positive density in the ductal cells. Likewise, in another experiment, systemic administration of endotoxin (LPS) for four times to KC mice also exacerbated overgrowth of pancreatic intraepithelial neoplasia and tubular and papillary glands (Figure 1E). These data demonstrated that elevated levels of systemic bile acids and LPS may accelerate the malignant transition into the PDAC state in a Kras background.

### 3.2. Oral Administration of Cationic Resin Attenuates Tumorigenesis and Metastasis through Sequestration of Intestinal Endotoxin and Bile Acids

**Figure 2:**
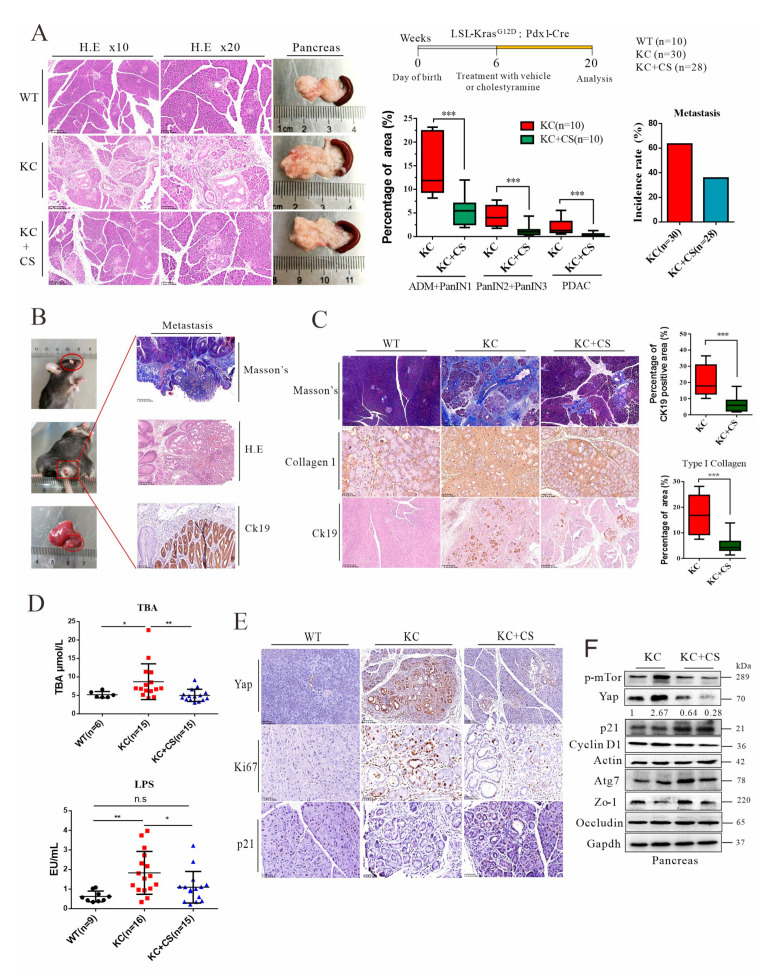
Oral administration of cationic resin attenuates tumorigenesis and metastasis through sequestration of intestinal endotoxin and bile acids. (**A**) Schematic presentation of cholestyramine treatment. Macroscopic images of pancreatic tissue of KC mice treated with vehicle or cholestyramine (CS). H&E scale bars = 250 μm (right) and 100 μm (middle). Quantitation of the percentages of early pancreatic lesions (ADM plus mPanIN1), late mPanIN lesions (PanIN2 plus PanIN3), and PDAC in mice treated with vehicle or CS. The metastasis incidence rate in mice treated with vehicle or CS. (**B**) Macroscopic image, histology and IHC staining of Ck19 in KC mouse metastasis. Masson’s trichrome staining scale bars = 500 μm (top), H&E scale bars = 250 μm (middle), Ck19 scale bars = 100 μm (bottom). (**C**) Masson’s trichrome staining (scale bars = 250 μm) and IHC staining for type 1 collagen (scale bars = 50 μm) and Ck19 (scale bars = 250 μm) in the pancreas. Quantitation of the percentage of type 1 collagen- and Ck19-positive areas. (**D**) Serum total bile acid levels and plasma endotoxin levels in WT and KC mice treated with vehicle or CS. Each point represented one individual sample. (**E**) IHC staining for Yap (scale bars = 100 μm), Ki67 (scale bars = 50 μm), and p21 (scale bars = 50 μm), as well as quantitation of Ki67 density. Representative images of histological staining and IHC staining are shown. (**F**) Western blot analysis of KC mouse pancreases after vehicle or CS treatment. * *p* < 0.05, ** *p* < 0.01, *** *p* < 0.001. The original Western Blot figures can be found in the Supplementary File S3.

Our previous work demonstrated that oral administration of cationic resin, such as cholestyramine, can sequester intestinal endotoxin (LPS), which consequently reduces systemic inflammation, improves insulin resistance, and relieves fatty liver and liver fibrosis [37,38]. Here, KC mice at 6 weeks of age were fed with or without cholestyramine in chow (3% *w*/*w*)—designated KC + CS and KC, respectively—for 14 consecutive weeks. As shown in Figure 2 and Figure S1, oral administration of cholestyramine significantly reduced PanIN sprouting, spontaneous bile duct obstruction rate, and metastasis rate in the liver, eye, and colon. However, administration of charge-neutral polystyrene (PS) had no effect on pancreatic cancer progression (Figure S2A). In line with our previous report, we confirmed that cationic resins, such as cholestyramine and polymyxin B resin, can effectively sequestrate LPS in vitro (Figure S2B). As anticipated, administration of cholestyramine significantly reduced the serum levels of total bile acids and plasma LPS levels in KC mice, which was further related to attenuation of systemic TNF-alpha levels in vivo (Figure 2D and Figure S2B,C). Although *Pdx-1* also expresses out of pancreatic, forced expression of *Kras^G12D^* in the liver or colon cannot induce carcinoma or ductal carcinoma [39,40]. Thus, the Ck19-positive cells in ductal neoplasia morphology that was observed in the extra-pancreatic tumor indicated that the metastasis originated from primary pancreatic ductal cells (Figure 2B). Tissue fibrosis in cancer is formed by necroinflammation and growth factors such as TGF-beta for wound healing. As shown in Figure 2C, less pancreatic fibrosis from administration of cholestyramine corroborated the reduction of CK19-positive cancerous areas. Notably, Ki67 density, an indicator of DNA replication, was also reduced by oral administration of cationic resin. Accordingly, Yap expression was downregulated by cholestyramine treatment, while p21 (cyclin-dependent kinase inhibitor 1) expression was upregulated, indicating that cholestyramine treatment and removal of systemic LPS and bile acids may suppress tumor progression in a specific mechanism (Figure 2E,F). Together, these results showed that oral administration of cationic resin can efficiently reduce the influx of intestinal LPS and bile acids, which consequently suppresses systemic inflammation and stress, leading to attenuation of pancreatic tumor growth and relieving of metastasis in PDAC.

### 3.3. Oral Administration of Cationic Resin Leads to Mobilization of Autophagic Flux for Yap Degradation in Pancreatic Cancer Tissues

**Figure 3:**
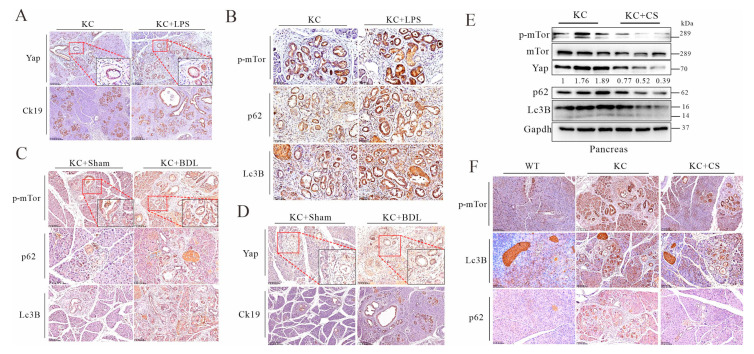
Oral administration of cationic resin leads to mobilization of autophagic flux for Yap degradation in pancreatic cancer tissues. Experimental conditions were described in Figure 1. (**A**) IHC staining of pancreatic tissue for Yap (scale bars = 100 μm) and Ck19 (scale bars = 250 μm) in KC mice treated with vehicle or LPS. (**B**) IHC staining of pancreatic tissue for p-mTor (scale bars = 50 μm), p62 (scale bars = 50 μm) and Lc3B (scale bars = 50 μm) in KC mice treated with vehicle or LPS. (**C**) IHC staining for p-mTor (scale bars = 100 μm), p62 (scale bars = 100 μm), and Lc3B (scale bars = 100 μm) in KC mice that received sham or BDL. (**D**) IHC staining for Yap (scale bars = 100 μm) and Ck19 (scale bars = 250 μm) in KC mice that received sham or BDL. (**E**) Western blot analysis of KC mouse pancreases treated with vehicle or cholestyramine (CS). (**F**) IHC staining for p-mTor, Lc3B, and p62 in WT mice and KC mice treated with vehicle or CS. Scale bars = 100 μm. The original Western Blot figures can be found in the Supplementary File S3.

Among the downstream targets of KRAS, the Hippo pathway with YAP and TAZ (YAP/TAZ) is crucial for cancer initiation and progression [29]. Studies showed that YAP is upregulated in PDAC in association with pancreatic fibrotic stiffness and poor prognosis of the patients [31,41]. We tested whether activation of autophagic flux could impact YAP turnover. In a cell culture experiment, depletion of amino acids promoted turnover of p62/SQSTM1 and LC3B, in line with YAP degradation, and chloroquine treatment impeded the autophagic flux, resulting in restoration of YAP (Figure S3A). Morphologically, amino acid depletion restored tight junctions and cellular quiescence, consistent with autophagic activation and YAP degradation (Figure S3B). Furthermore, knockdown of ATGs could restore YAP levels as well (Figure S3C). Endotoxin is known for its promotion of fibrotic stiffness [38,42], and here we noticed that additional treatment with LPS promoted nuclear localization of Yap in *Pdx1-Kras* mice, in agreement with increased pancreatic fibrosis and sprouting of ductal neoplasia (Figure 3A). The accumulation of Yap in KC mice treated with LPS was also related to increased phosphorylation of mTor and slowed turnover of p62/Sqstm1 and Lc3B, showing impairment of autophagic flux leading to Yap degradation (Figure 3A,B). Likewise, bile ductal ligation and induced reflux generated the same phenotypic results as LPS treatment (Figure 3C,D). Administration of cholestyramine, presumably through depletion of intestinal endotoxin and bile acids, resulted in downregulation of Yap in agreement with accelerated autophagic-lysosomal flux and accelerated turnover of p62/Sqstm1 in *Pdx1-Kras* mice, which is related to the reduced phosphorylation and inactivation of mTor (Figure 3E,F).

### 3.4. Autolysosomal Stress Induced by Chloroquine Treatment Accelerates Malignant Progression of Pancreatic Cancer in Mice

**Figure 4:**
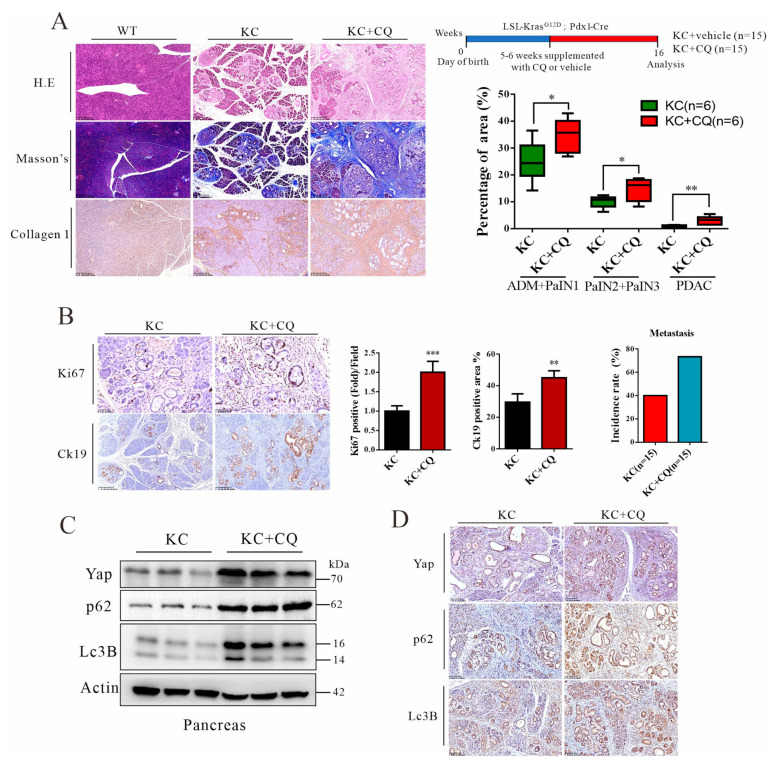
Autolysosomal stress induced by chloroquine promotes malignant progression of pancreatic cancer in KC mice. (**A**) A scheme presents the treatment protocol. KC + vehicle (*n* = 15) and KC + CQ (*n* = 15). Histology and IHC staining of type 1 collagen in pancreatic tissue (scale bars = 250 μm) and quantitation of the percentages of pancreatic lesions as well as the type 1 collagen-positive area, *n* = 6. (**B**) IHC staining for Ki67 (scale bars = 50 μm) and Ck19 (scale bars = 250 μm) as well as quantitation of the Ck19-positive area, Ki67 density, and metastasis incidence rate. (**C**) Western blot analysis of KC mouse pancreases treated with vehicle or CQ. (**D**) IHC staining for Yap, p62, and Lc3B of the pancreatic tissues. Scale bars = 100 μm. Representative images of IHC staining were shown. * *p* < 0.05, ** *p* < 0.01, *** *p* < 0.001. The original Western Blot figures can be found in the Supplementary File S3.

The notion of lysosomal dysfunction in tumorigenesis is emerging. For example, one study showed that *Drosophila melanogaster* harboring a human *H-Ras* gene was not sufficient for tumorigenesis, but additional disruption of a gene for lysosomal biogenesis, called *dor*, or feeding the *H-Ras* transgenic fly with chloroquine was needed, suggesting that lysosomal flux may promote malignancy [43]. To this end, we created systemic lysosomal stress by adding chloroquine in drinking water at 2 mg/mL to KC mice for 16 consecutive weeks. Consequently, tumor progression at all stages—ranging from PanIN to PDAC—was accelerated by chloroquine treatment, which is further in line with the increased level of pancreatic fibrosis (Figure 4A). Similarly, periodontal injection of chloroquine (60 mg/kg body weight) also provoked malignant progression (Figure S4). Lysosomal stress significantly increased the rate of metastasis (Figure 4B and Figure S4B). Similarly, ductal hypertrophy was correlated with Ck19- and Ki67-positive stained in the ductal cells, which is further in agreement with lysosomal stress (Figure 4C and Figure S4). Importantly, autolysosomal stress was evident by chloroquine treatment, showing accumulation of p62/Sqstm1 and failed conversion of Lc3B, leading to Yap upregulation (Figure 4D,E). Taken together, these results indicate autolysosomal flux in the cancer cells may suppress malignant progression.

### 3.5. Cystatin A, an Endogenous Inhibitor of Lysosomal Acidic Proteinases, Is Upregulated in PDAC Patients and KC Mice and Is Associated with Poor Prognosis

**Figure 5:**
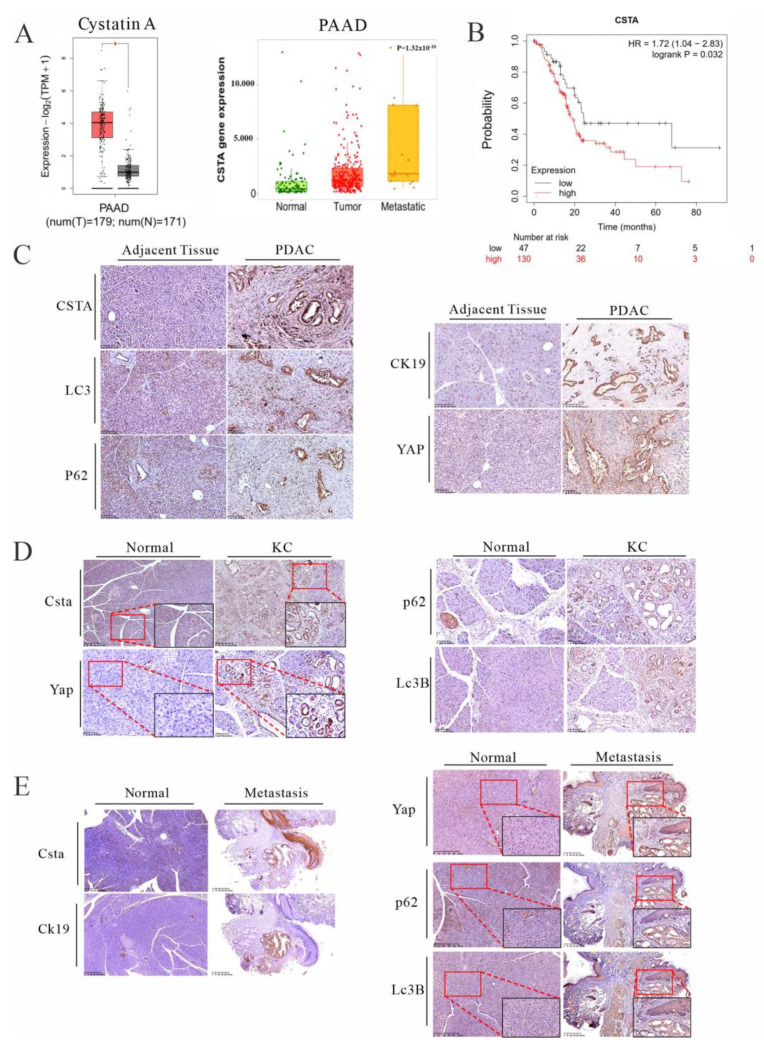
A high level of cystatin A expression, an indication of lysosomal stress, is associated with malignant progression of pancreatic cancer. (**A**) Boxplot analysis (left) of *CSTA* expression in pancreatic tumors (T) and normal regions (N) based on the GEPIA2 database (http://gepia2.cancer-pku.cn/, 19 December 2021). Comparison of *CSTA* expression profiles (right) in normal, pancreatic adenocarcinoma (PAAD), and metastatic tissues based on the TNMplot database (https://tnmplot.com/analysis/, 19 December 2021). (**B**) Kaplan–Meier survival analysis of *CSTA* expression in pancreatic cancer based on the Kaplan–Meier Plotter database (http://kmplot.com/analysis/, 19 December 2021). (**C**) IHC staining of human pancreatic tissues of normal adjacent and PDAC specimens for CSTA (scale bars = 100 μm), YAP (scale bars = 100 μm), P62 (scale bars = 100 μm), LC3 (scale bars = 100 μm), and CK19 (scale bars = 250 μm). (**D**) IHC staining for Csta (scale bars = 250 μm), Yap (scale bars = 100 μm), p62 (scale bars = 100 μm), and Lc3B (scale bars = 100 μm) in the pancreas of normal and KC mice. (**E**) IHC staining for Csta, Ck19, Yap, p62, and Lc3B in normal mouse pancreas and KC mouse metastasis. Scale bars = 250 μm. Representative images of IHC staining were shown.

Cellular inhibitors of lysosomal acidic proteinases are critical for the determination of the digestive flux of lysosomes. We retrieved public databases (GEPIA 2: http://gepia2.cancer-pku.cn, accessed on 19 December 2021 and TNMplot: https://tnmplot.com/analysis/, accessed on 19 December 2021) and noticed that cystatin A (CSTA) expression was significantly increased in PDAC patients at the metastasis phase (Figure 5A). Moreover, PDAC patients who had higher levels of CSTA were associated with poor overall survival (Figure 5B). In contrast, high expression of cathepsin L (CTSL), a major lysosomal acidic proteinase that can be directly inhibited by CSTA, was positively associated with overall survival (Figure S5A). We also examined human specimens of PDAC and found markedly upregulated CSTA expression in ductal cells in pancreatic tissues, and this notion was further supported by data from the Human Protein Atlas and clinical data [44] (Figure 5C and Figure S5B). Importantly, along with elevated expression of CSTA, the autophagic substrates p62/SQSTM1, LC3B, and YAP all accumulated in the ductal epithelia, showing impaired autophagic flux in the pancreatic tissues of PDAC patients (Figure 5C). Finally, we examined this notion in our KC mice, which gave a very similar pattern of pathophysiological findings, showing high level expression of CstA in association with impeded lysosomal turnover of p62/sqstm1 and Lc3B, leading to Yap accumulation (Figure 5D,E). Thus, these results demonstrate that lysosomal stress and impairment of its flux, in part through the increased expression of cystatins, may ultimately lead to YAP accumulation for tumor growth and metastatic dissemination.

### 3.6. Forced Expression of Cystatin A and Lysosomal Stress Leads to YAP Accumulation in Cancer Cells

**Figure 6:**
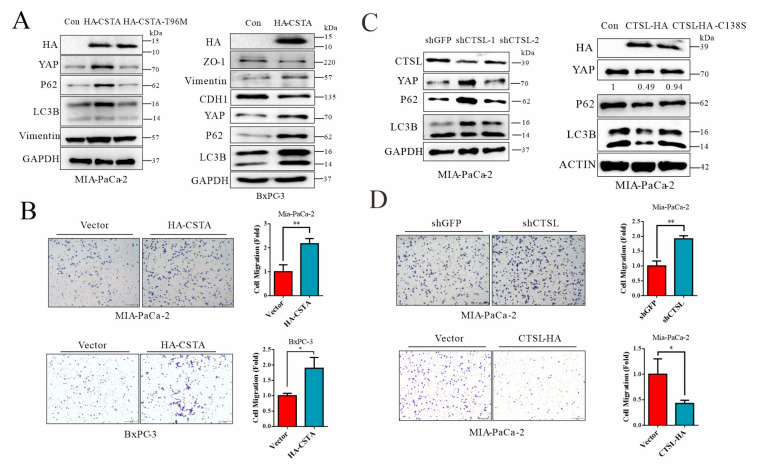
Forced expression of cystatin A leads to lysosomal stress and failed autolysosomal flux, leading to YAP accumulation in cancer cells. (**A**) Western blot analysis of BxPC-3 and MIA-PaCa-2 cells overexpressing CSTA. (**B**) Transwell analysis of MIA-PaCa-2 and BxPC-3 cells with vector or CSTA overexpression. Scale bars = 200 μm. (**C**) Western blot analysis of MIA-PaCa-2 cells with shRNA-based knockdown of CTSL (top). Western blot analysis of MIA-PaCa-2 cells overexpressing CTSL or CTSL mutations (bottom). (**D**) Transwell analysis of MIA-PaCa-2 with shRNA-based knockdown of CTSL (top). Transwell analysis of MIA-PaCa-2 cells overexpressing CTSL (bottom). Scale bars = 200 μm. * *p* < 0.05, ** *p* < 0.01. The original Western Blot figures can be found in the Supplementary File S3.

Through an in vitro experiment, we further validated the role of the endogenous inhibitor of lysosomal cathepsins, CSTA, and autolysosomal flux for YAP turnover. First, we forcedly expressed CSTA in two human PDAC cell lines. As shown in Figure 6A, overexpression of CSTA in BxPC3 cells led to autolysosomal stress, showing failed turnover of p62/SQSTM1 and LC3B, resulting in YAP accumulation. In MIA-PaCa-2 cells, the expression of wild-type but not inactive cystatin A (CSTA/T96 M) suppressed autolysosomal flux and restored YAP levels. Transwell migration assays showed that forced expression of CSTA promoted cell migration, in agreement with YAP stabilization and enhanced expression of the intermediate filament of vimentin for cellular motility (Figure 6B). Likewise, knockdown of lysosomal cathepsin, CTSL, led to impairment of autolysosome flux, showing accumulation of p62/SQST1, LC3B, and YAP and enhanced cell migration (Figure 6C,D). The opposite is also true, since overexpression of cathepsin L increased autolysosome flux and enhanced turnover of p62/SQSTM1, LC3B, and YAP, which is in line with reduced cell dissemination (Figure 6C,D). Thus, these results indicate that lysosomal stress and impairment of autolysosome flux can result in YAP accumulation and cancer cell dissemination.

### 3.7. Chenodeoxycholic Acid and LPS Can Activate the AKT-mTOR Pathway, Leading to Impairment of Autophagic Flux and YAP Accumulation in Cancer Cells

**Figure 7:**
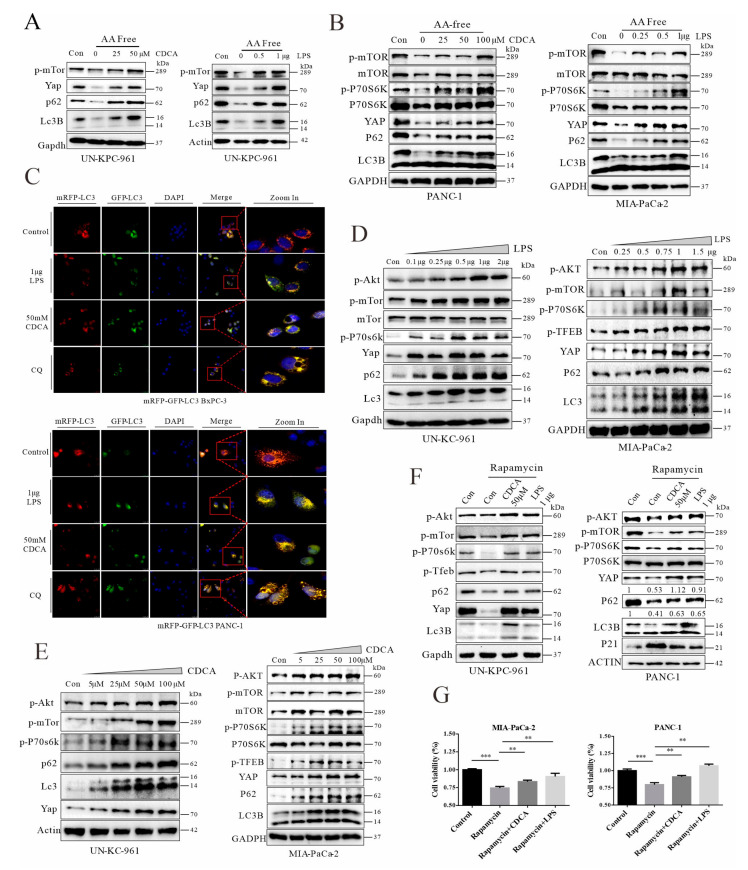
Chenodeoxycholic acid and LPS can activate the AKT-mTOR pathway, leading to impairment of autophagic flux and YAP accumulation in cancer cells. (**A**) Western blot analysis of UN-KPC-961 cells treated with CDCA or LPS in the condition of amino acid depletion for 12 h. In order to mobilize autolysosomal flux. (**B**) Western blot analyses of PANC-1 and MIA-PaCa-2 cells treated with CDCA and LPS, respectively, under starvation for 12 h. (**C**) Florescent microscopy of RFP-GFP-LC3 analyses in BxPC-3 and PANC-1 cells treated with vehicle, 50 μM CDCA, 1 μg LPS, and 20 μM CQ. (**D**) Western blot analyses of UN-KC-961 and MIA-PaCa-2 cells treated with different concentrations of LPS. (**E**) Western blot analyses of UN-KC-961 and MIA-PaCa-2 cells treated with different concentrations of CDCA for 12 h. (**F**) Western blot analyses of UN-KPC-961 and PANC-1 cells treated with LPS or CDCA under 100 μM rapamycin treatment for 12 h. (**G**) Cell viability analysis of MIA-PaCa-2 and PANC-1 cells treated with 0.5 μg LPS or 50 μM CDCA under 200 nM rapamycin treatment. Representative images of experiments repeated 2–3 times. ** *p* < 0.01, *** *p* < 0.001. The original Western Blot figures can be found in the Supplementary File S3.

Increased levels of endotoxin and reflux of bile acids are the major consequences of biliary obstruction [45], while chenodeoxycholic acid is a major component of bile. A pharmacologic study found that cholestyramine binds preferentially to hydrophobic acid chenodeoxycholic acid over the hydrophilic cholic acid [46], and it was demonstrated that chenodeoxycholic acid levels could be significantly reduced by cholestyramine intervention [47]. Through in vitro experiments, we further investigated the direct impact of bile acids and LPS on autophagic flux and Yap degradation. In murine cells derived from *Pdx1-Kras* mice and two human PDAC cell lines, autolysosomal flux was accelerated through depletion of amino acids in the culture medium. Conversely, treatment with chenodeoxycholic acid (CDCA) or LPS impaired autolysosomal flux, showing failed turnover of p62/SQSTM1 and LC3B and consequent accumulation of YAP (Figure 7A,B). The RFP-GFP-LC3 assay in living cells was used to determine the autolysosomal flux in situ and impact of CDCA and LPS treatments. Autolysosomal flux was indicated by the degradation of an acidic-pH-sensitive variant of GFP-LC3. As shown in Figure 7C and Figure S6A, CDCA or LPS treatment impaired autolysosomal flux, showing accumulation of GFP-LC3 and yellow puncta. Furthermore, CDCA and LPS treatments activated the AKT-mTOR pathway, showing increased phosphorylation of p-AKT, p-mTOR, p-TFEB, and p-P70S6K (Figure 7D,E, and Figure S6B), which explains the inhibition of autolysosomal flux. Likewise, rapamycin is known for its inhibition of mTOR and activation of autolysosomal flux. Here, we found that CDCA and LPS could antagonize the rapamycin-mediated suppression of the AKT/mTOR pathway (Figure 7F). Moreover, the rapamycin-mediated suppression of cell viability could be blunted by CDCA and LPS treatments (Figure 7G).

### 3.8. Signals from FXR and TLR4 Mediate Chenodeoxycholic Acid and LPS, Respectively, for Activation of AKT-mTOR Pathway in Pancreatic Cancer Cells

**Figure 8:**
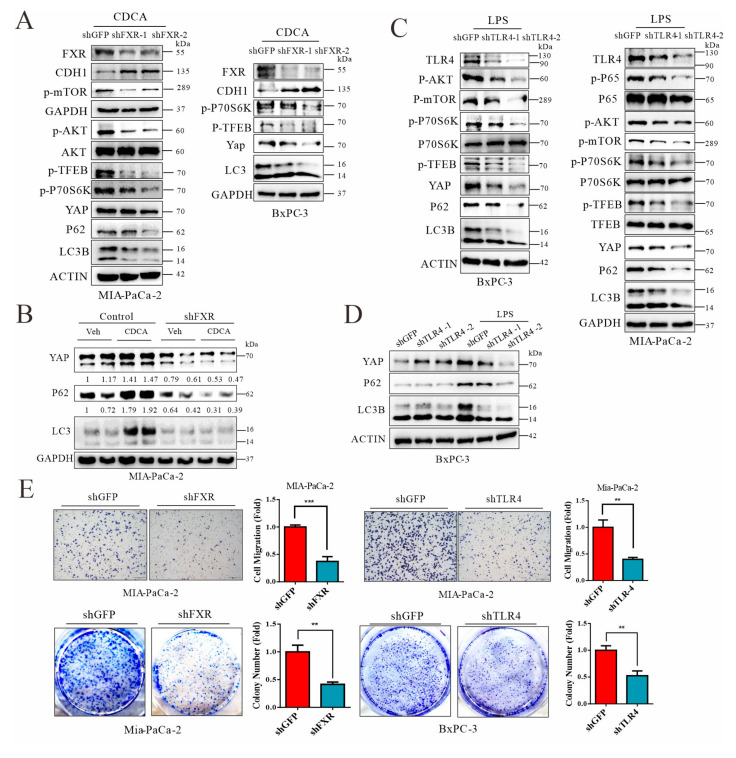
Signals from FXR and TLR4 mediate chenodeoxycholic acid and LPS, respectively, for activation of AKT-mTOR pathway in pancreatic cancer cells. (**A**) Western blot analysis of Mia-PaCa-2 and BxPC3 cells subjected to shRNA-based knockdown of FXR that were treated with 50 μM CDCA for 12 h. (**B**) Western blot analysis of autophagic flux for YAP degradation in MIA-PaCa-2 cells with FXR knockdown. (**C**) Western blot analysis of Mia-PaCa-2 and BxPC3 cells subjected to TLR4 knockdown and treated with 0.5 μg LPS for 12 h was performed. (**D**) Western blot analysis of autophagic flux for YAP degradation in BxPC3 cells subjected to TLR4 knockdown. (**E**) Transwell analysis and colony formation analysis of MIA-PaCa-2 cells with knockdown of FXR or TLR4 and treated with 50 μM CDCA or 0.5 μg LPS. Representative images of experiments repeated 2–3 times. ** *p* < 0.01, *** *p* < 0.001. The original Western Blot figures can be found in the Supplementary File S3.

**Figure 9:**
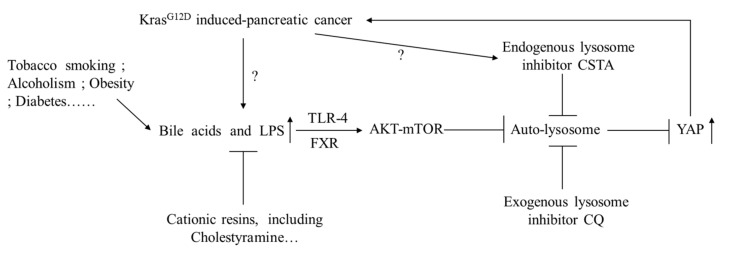
Hypothetical figure. *Kras^G12D^* mutation and pancreatic cancer risk factors, such as obesity and diabetes, can induce hyperbileacidemia and hyperendotoxemia, which promote pancreatic cancer progression via inhibiting autophagic flux for YAP degradation. Cationic resins like cholestyramine can restrict pancreatic cancer progression by sequestering intestinal acidic toxins. CQ: chloroquine; FXR: Farnesoid X receptor; TLR-4: Toll-like receptor 4; CSTA: Cystatin A; YAP: Yes-associated protein.

In animal work, we noted that cholestyramine treatment reduced the nuclear localization of Fxr in cancerous pancreatic ductal cells and downregulated p65, a Tlr-4 downstream factor (Figure S7). To this end, we knocked down two respective receptors and explored the signaling pathway involved in the suppression of autophagic flux. As shown in Figure 8A,B, knockdown of FXR in human pancreatic cancer cells suppressed activation of the AKT-mTOR pathway. Knockdown of FXR also abolished the ligand-induced suppression of autophagic flux and restored YAP degradation. Similarly, TLR4 knockdown antagonized LPS-induced AKT-mTOR activation and enhanced autolysosome flux (Figure 8C,D). Transcription factor EB (TFEB) promotes the expression of many lysosomal and autosomal factors [48]. As a downstream target of mTOR through phosphorylation, TFEB is also regulated by FXR, since knockdown of the nuclear receptor leads to reduced phosphorylation of TFEB, which may further promote lysosomal biogenesis. Likewise, knockdown of TLR4 resulted in autophagic flux for YAP degradation, presumably through failed activation of AKT-mTOR. Finally, knockdown of FXR and TLR4 also restricted the capacity of colony formation and cell migration (Figure 8E). Thus, mechanistically, elevated levels of chenodeoxycholic acid and LPS in biliary obstruction and intestinal impairment can activate the AKT/mTOR pathway, leading to TFEB inhibition and lysosomal stress for YAP accumulation in tumorigenesis and metastasis. A hypothetical figure was provided in Figure 9.

## 4. Discussion

Genetic acquisition of Kras and gain-of-function mutation in pancreatic ductal cells may occur in an early life stage, but it takes decades for full malignant onset, leading to PDAC, indicating that additional hits are essential. In contrast to other cancers of the digestive system, such as hepatocellular carcinoma (HCC) which occurs at middle age, PDAC is often undetected until old age approximately 65–70 years old [49]. The age-delayed onset of PDAC is associated with the nature of extreme softness of the pancreas. The elastic modulus for healthy pancreas is at approximately 1.0–1.5 kPa [50]. Conversely, in advanced PDAC, which is often associated with pancreatic fibrosis, stiffness can reach 3.8 kPa [50]. On the other hand, the liver is a stiff organ with an elastic modulus of 6 kPa, and liver cancer often occurs at middle age in association with accelerated stiffness at 8–12 kPa [51]. In fact, most risk factors for PDAC—such as tobacco smoking, alcohol abuse, and chronic pancreatitis—cause pancreatic fibrosis and stiffness [52]. Tissue fibrosis and stiffness in the pancreas are mostly mediated by the activation of pancreatic stellate cells for ECM synthesis and crosslinking, which is largely driven by chronic inflammation that orchestrates wound healing and fibrotic stiffness. In terms of PDAC, YAP activation can activate pancreatic fibroblasts, promoting PC progression, whereas inhibiting YAP expression prevents tumor growth and fibrosis formation [53]. Additionally, YAP is also notorious for its role in promoting tumorigenesis and metastasis, thus regulation of YAP expression can be considered as a potential method of cancer treatment.

On the other hand, risk factors for tumorigenesis—such as smoking, alcoholism, diabetes, and obesity—are associated with gut dysbiosis [12,54]. Excessive and persistent commensal microbe debris and decomposition are generally toxic to the host and can cause chronic inflammation that consequently leads to insulin resistance, metabolic disorders, and tissue fibrosis [55,56,57]. We realized that many of the toxins from gut—such as endotoxin (LPS), certain flagellins, and bacterial CpG-DNA—are negatively charged acids in their chemical nature, which serve as ligands for pattern recognition receptors (PPRs). Indeed, persistent and excessive activation of PPR pathways can compromise host immunity to tumorigenesis [58,59]. Thus, we predict that sequestration excretion of these acidic PPR ligands through non-digestible cationic polymers or resins (anionic exchange resins) may consequently ameliorate the systemic and hepatic inflammation, and autophagic stress, which can ultimately be used as a new strategy for cancer prevention and treatment.

Elevated levels of LPS are often present in the pancreatic tissues of PDAC, in association with poor prognosis and loss of therapeutic efficacy of gemcitabine [18]. Increased plasma levels of LPS are related to enrichment in Proteobacteria, a major phylum of Gram-negative bacteria including *E. coli* [13,60]. One study found that pathogenic *Enterococcus* and capsular polysaccharide levels were increased in pancreatitis and pancreatic cancer patients [61]. Similarly, a high level of plasma endotoxin is related to advanced liver cancer [62]. Mechanistically, injured intestinal epithelia and gut permeability were demonstrated in animal models of pancreatic cancer in association with increased endotoxemia [12,63,64]. While the role of endotoxins in promoting inflammation and cancer is well established, the direct evidence for LPS in promoting cancer formation and metastasis is surprisingly inadequate. In this study, and with animal models, we confirmed the strong association of elevated levels of endotoxin with the malignant transformation of pancreatic cancer in a Pdx1-Kras background. In vitro, knockdown of the LPS receptor TLR-4 in pancreatic cancer cell lines dramatically decreased Yap protein levels while in the presence of autophagic stress. Importantly, we showed that oral administration of cationic resins such as cholestyramine can reduce plasma levels of LPS to ameliorate systemic inflammation, which ultimately suppresses cancer metastasis. These results indicate that sequestration and excretion of intestinal endotoxin may be applied to prevent and treat pancreatic malignant transformation.

Biliary obstruction and its associated reflux of bile acids in line with jaundice are common in later stages of pancreatic patients, and animal work showed that bile reflux into the pancreatic ducts promotes metastasis of PDAC [21,65]. A study revealed that bile acids promoted PDAC progression through induction of Muc4 expression [66]. Neonatal injury of pancreatic beta cells by streptozotocin (STZ) followed by feeding with a high-fat diet could induce spontaneous hepatocellular carcinoma (HCC) in line with intrahepatic retention of hydrophobic bile acids—including deoxycholate (DCA), taurocholate (TCA), etc.—while administered cholestyramine could ameliorate HCC in the mice [67]. High expression of the hydrophobic bile acid receptor FXR and TGR5 in pancreatic cancer patients was associated with poor prognosis [22,68]. Moreover, activation of FXR and TGR5 in pancreatic cells mediated pancreatic cellular injury [69,70]. In this study, we also demonstrated that biliary obstruction and bile reflux can promote the malignancy of PDAC in animal models. Importantly, cholestyramine-mediated amelioration of PDAC is related to improved autophagy-lysosomal flux in pancreatic cells, leading to Yap downregulation. Mechanistically, knockdown of the hydrophobic bile acid receptor FXR in pancreatic cell lines significantly reduced Yap accumulation induced by autophagy inhibition as well as cell growth and migration. Additionally, nuclear FXR expression was also reduced after cholestyramine intervention in the Pdx1-Kras mouse model, indicating that FXR activation was involved in pancreatic cancer progression.

Autophagy is a cellular mechanism in catabolism and organelle clearance, while its roles in cancer are controversial, seemingly depending on cancer stages and cancer types. Intervention or inhibition of autophagy has been attempted as an anti-cancer therapy because several studies found some cancers apply autophagy to escape external stress like hypoxia, chemotherapy, or radiotherapy [71,72]. Several groups recently discovered that autophagy was required by cancer cells to protect against lethal metabolic stresses and maintain metabolic homeostasis in RAS-driven tumorigenesis [73,74,75]. On the other hand, oncogenic RAS can cause senescence and cell death via several essential autophagy proteins [76,77]. Moreover, clinical trials using lysosome inhibitors, such as hydroxychloroquine, failed to improve the survival rate of pancreatic cancer [78,79]. Mechanistically, transition from PanIN to PDAC was accompanied by genetic alteration including KRAS, TP53, and CDKN2A [80]. Of note, a recent study pointed out that autophagic flux serves as a gate-keeper for genome instability, limiting genetic mutation accumulation and implying that autophagic flux can suppress pancreatic cancer progression [81]. As a cellular degradation and recycling center, the lysosome is a metabolic signaling hub essential for metabolism, cell growth, and differentiation [82]. The notion that lysosomal dysfunction causes tumorigenesis is emerging. Likewise, knockout of the lysosomal cathepsin L gene (CtsL) in mice exacerbates tumor growth in the epidermis of mice expressing the human papillomavirus oncogene K14–HPV16 [83]. Cystatins, endogenous inhibitors of lysosomal acidic proteinases, are often overexpressed in association with poor prognosis in cancers [84,85]. Lysosome-based degradation relies on autophagic activation and digestive flux. Indeed, impairment of autophagy—such as genetic disruption of beclin-1—can cause tumorigenesis in animal models [86], and knockdown of Atg5 promotes KRas-mediated PDAC [87]. In this study, we discovered a link among pancreatic fibrosis, malignance, and impaired autophagy-lysosomal flux, as evidenced by decreased turnover of p62/Sqstm1 and increased expression of cystatin A, an endogenous inhibitor for lysosomal cysteine cathepsin H/L. Hippo-mediated phosphorylation and subsequent ubiquitination of YAP was proposed as a mechanism for YAP degradation [88]. However, our team and other researchers have noticed that autophagy activation in many ways can promote Yap degradation [89,90]. For instance, removal of amino acids from the culture medium could promptly activate autophagic flux and promote Yap degradation, which is impeded by lysosomal stress caused by chloroquine or forced expression of cystatin A.

## 5. Conclusions

In conclusion, the present work found that hyperbileacidemia and hyperendotoxemia were related to tumorigenesis and the transition from PanIN to PDAC. Importantly, gut toxins can activate the AKT-mTOR pathway, leading to suppression of autophagic-lysosomal flux, which consequently can attenuate YAP degradation. The roles of acidic toxins in tumorigenesis are demonstrated here in a PDAC model, while similar mechanisms may be extended to other digestive cancers and even beyond, since gut dysbiosis and intratumor microbes are commonly related to the prognosis of many types of cancers. Finally, this study demonstrated a potential clinical application through sequestration of gut toxins by cationic resins—such as cholestyramine or colesevelam—for the prevention or treatment of various types of cancers.

## Data Availability

Materials are available on request.

## Supplementary Materials

The following supporting information can be downloaded at: https://www.mdpi.com/article/10.3390/cancers14061407/s1. File S1, Supplementary Figures S1–S7. Figure S1: KC mice spontaneously developed common bile duct obstruct; Figure S2: Cholestyramine can sequestrate LPS in vitro and reduced inflammatory factor expression in vivo; Figure S3: Autophagic flux regulated YAP degradation; Figure S4: Chloroquine injection worsen pancreatic cancer progression; Figure S5: *CTSL* expression data in KMP website and Cystatin A expression data from Human Protein Atlas website; Figure S6: Effects of LPS and CDCA on autophagic flux and AKT-mTOR pathway in pancreatic cells; Figure S7: FXR and p-p65 expression in KC mice treated with cholestyramine; File S2. Details of reagents, plasmids, primers, and antibodies; File S3. Original images of Western bolt analysis.

## Abbreviations

KC mice: *Pdx1-Cre:SL-Kras^G12D/+^* mice; CS: Cholestyramine; PanIN: Premalignant lesion of pancreatic intraepithelial neoplasia; PDAC: Pancreatic ductal adenocarcinoma; BDL: Common bile duct ligation; LPS: Lipopolysaccharide; CQ: Chloroquine; TBA: Total bile acid; YAP1: Yes-associated protein 1; mTOR: Mammalian target of rapamycin; H&E: Hematoxylin and eosin; CDCA: Chenodeoxycholic acid; CSTA: Cystatin A; ADM: Acinar-to-ductal metaplasia; CTSL: Cathepsin L.
