# Supplementary material for: Sequestration of Intestinal Acidic Toxins by Cationic Resin Attenuates Pancreatic Cancer Progression through Promoting Autophagic Flux for YAP Degradation
Source: Cancers (Basel). 2022 Mar 10;14(6):1407. doi: 10.3390/cancers14061407 (PMC8946475; doi:10.3390/cancers14061407)

Figure S2F

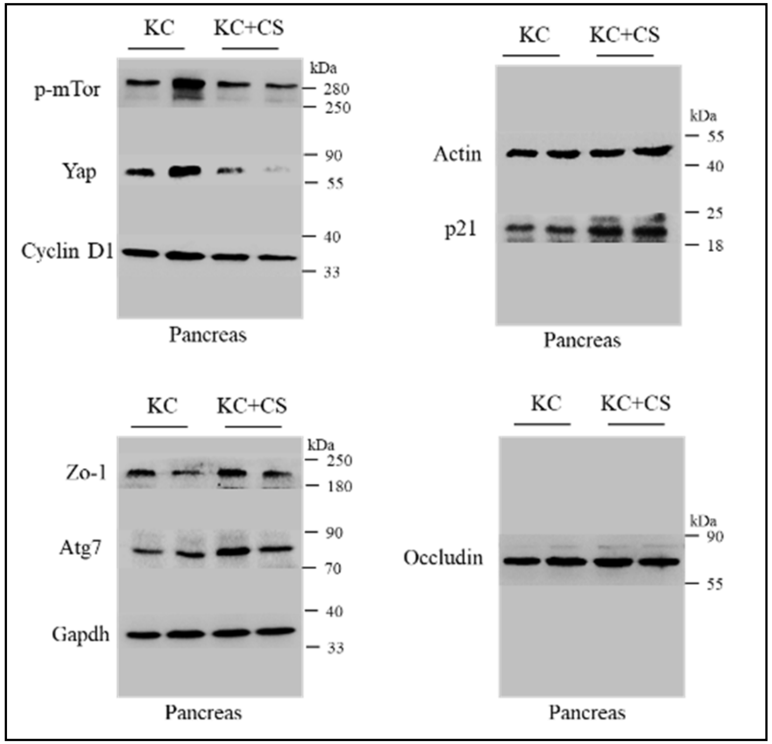

Figure S3E

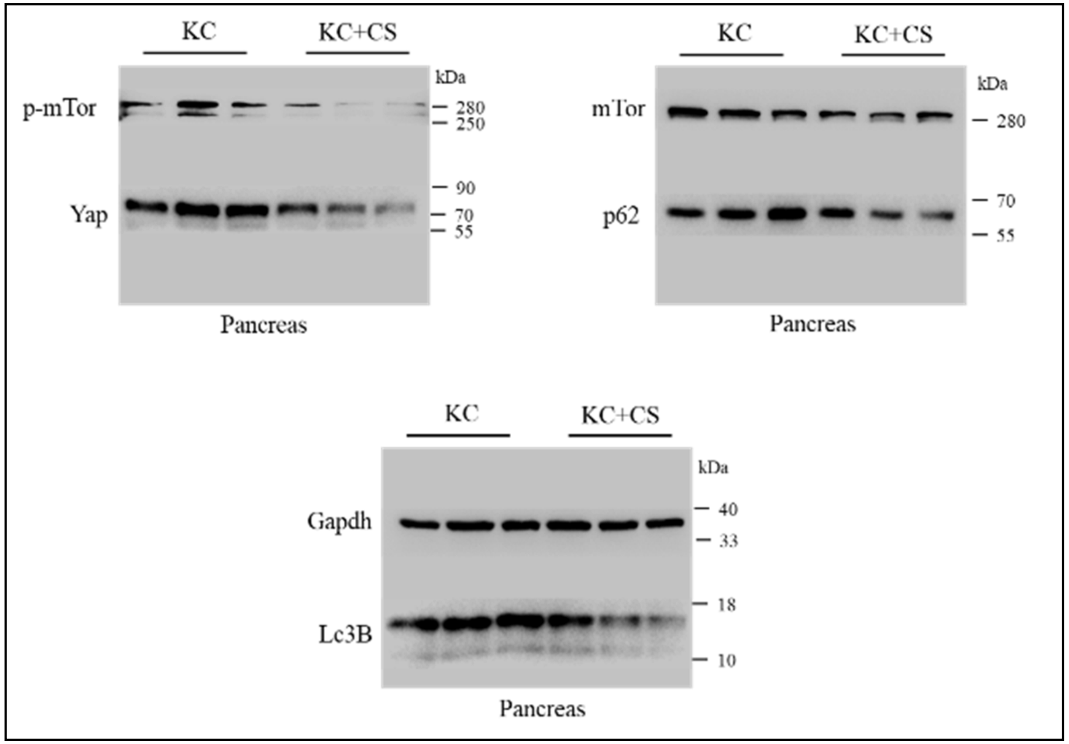

Figure S4C

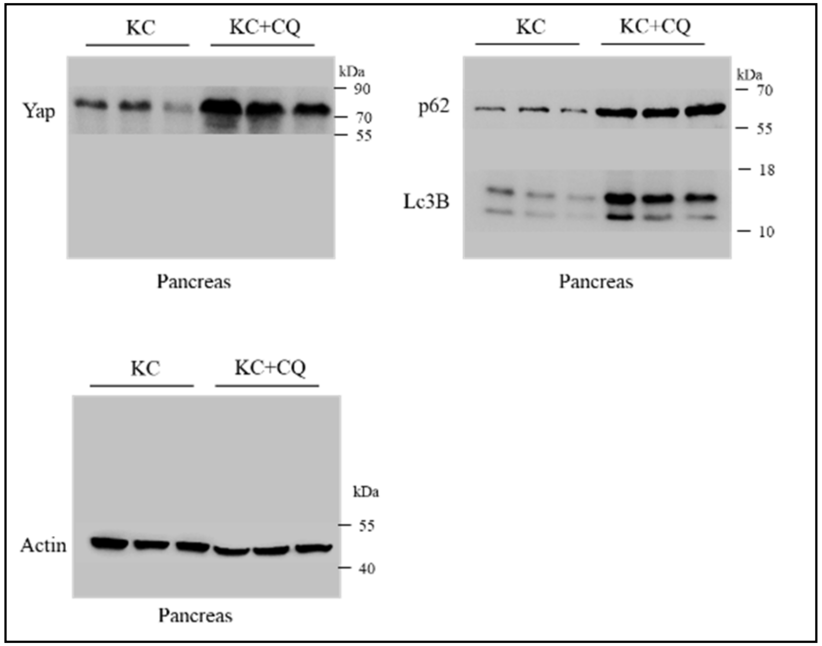

Figure S6A

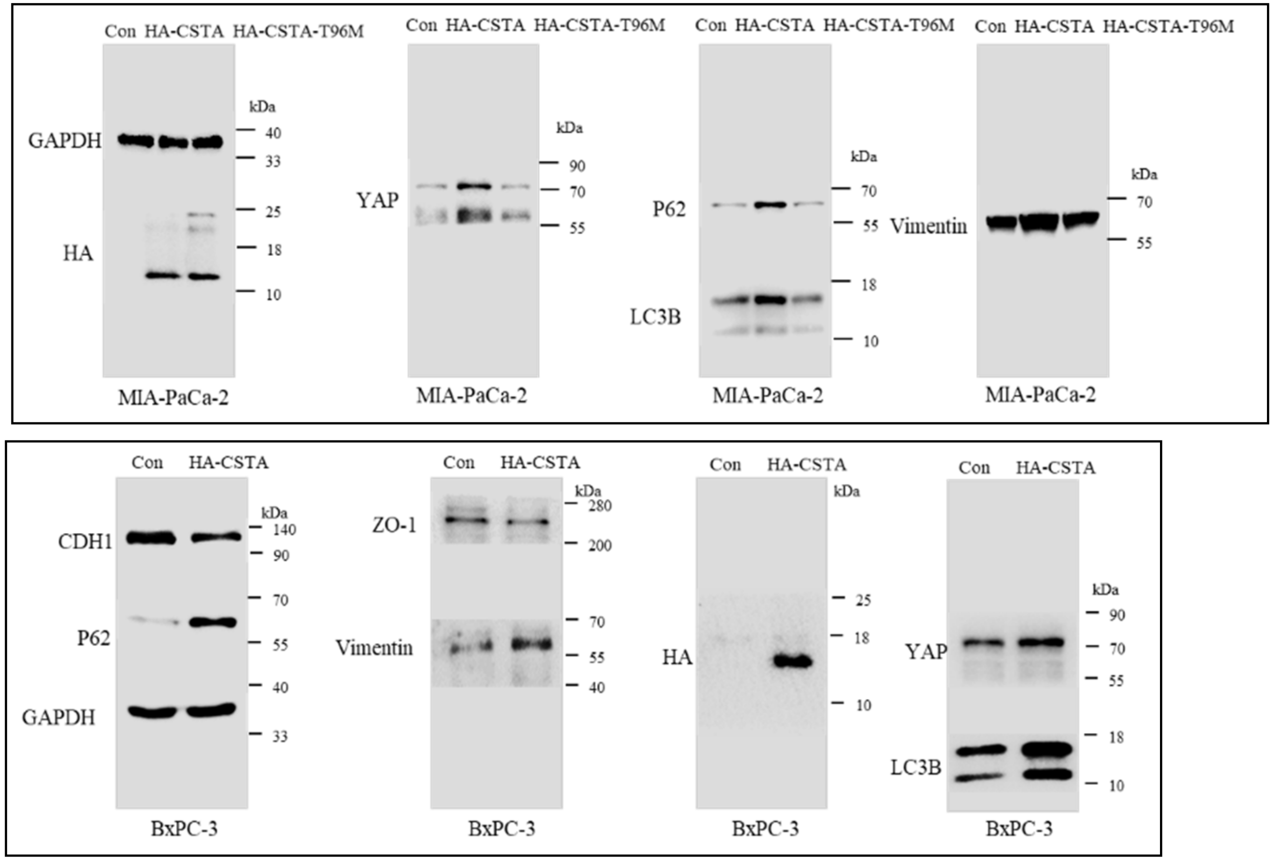

Figure S6C

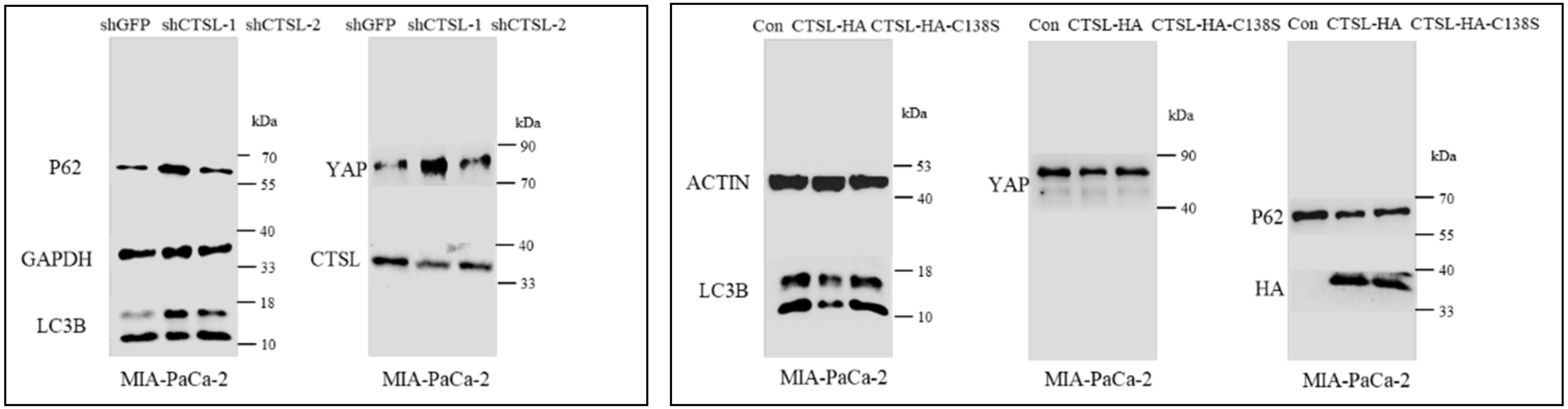

Figure S7A

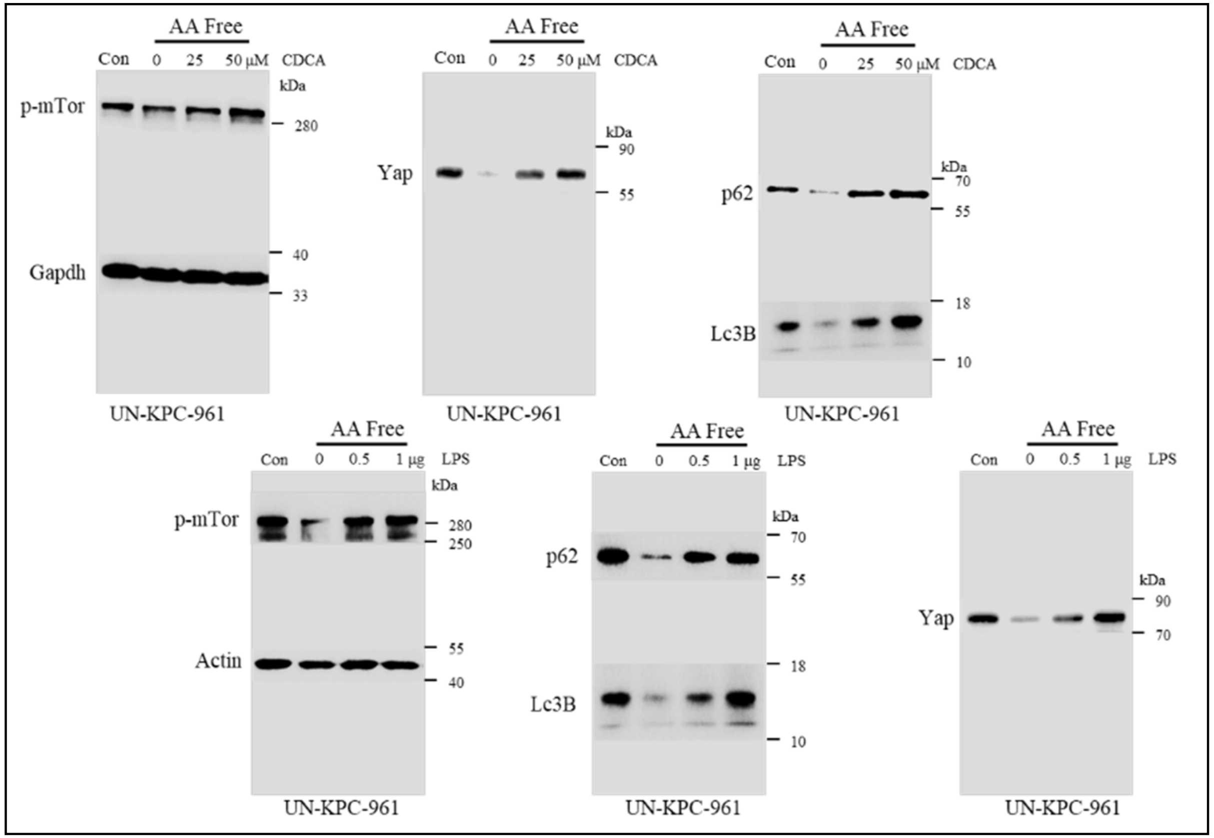

AA Free
Con 0 25 50 μM CDCA
kDa
p-mTor
280
Gapdh
40
33
UN-KPC-961
AA Free
Con 0 25 50 μM CDCA
kDa
90
Yap
55
UN-KPC-961
AA Free
Con 0 25 50 μM CDCA
kDa
70
p62
55
18
Lc3B
10
UN-KPC-961
AA Free
Con 0 0.5 1 μg LPS
kDa
p-mTor
280
250
Actin
55
40
UN-KPC-961
AA Free
Con 0 0.5 1 μg LPS
kDa
70
p62
55
18
Lc3B
10
UN-KPC-961
AA Free
Con 0 0.5 1 μg LPS
kDa
90
Yap
70
UN-KPC-961

Figure S7B

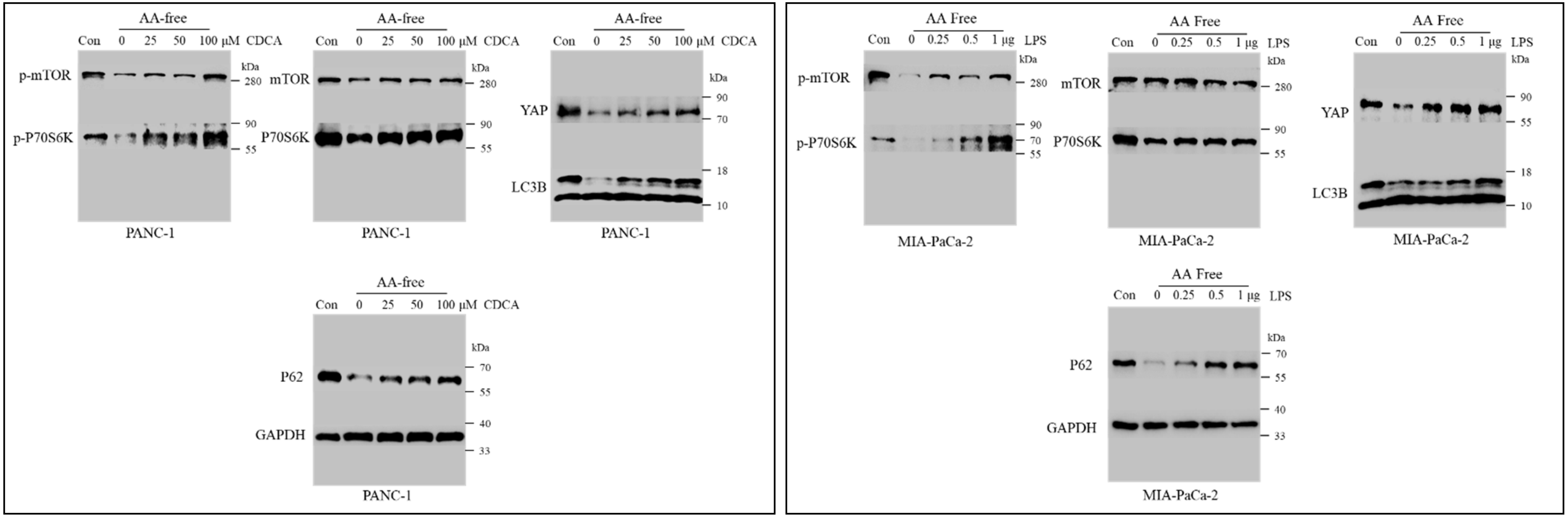

AA-free
Con
0
25
50
100 μM
CDCA
kDa
p-mTOR
p-P70S6K
mTOR
P70S6K
YAP
LC3B
P62
GAPDH
280
90
70
55
40
33
18
10
PANC-1
AA Free
0.25
0.5
1 μg
LPS
MIA-PaCa-2

Figure S7D

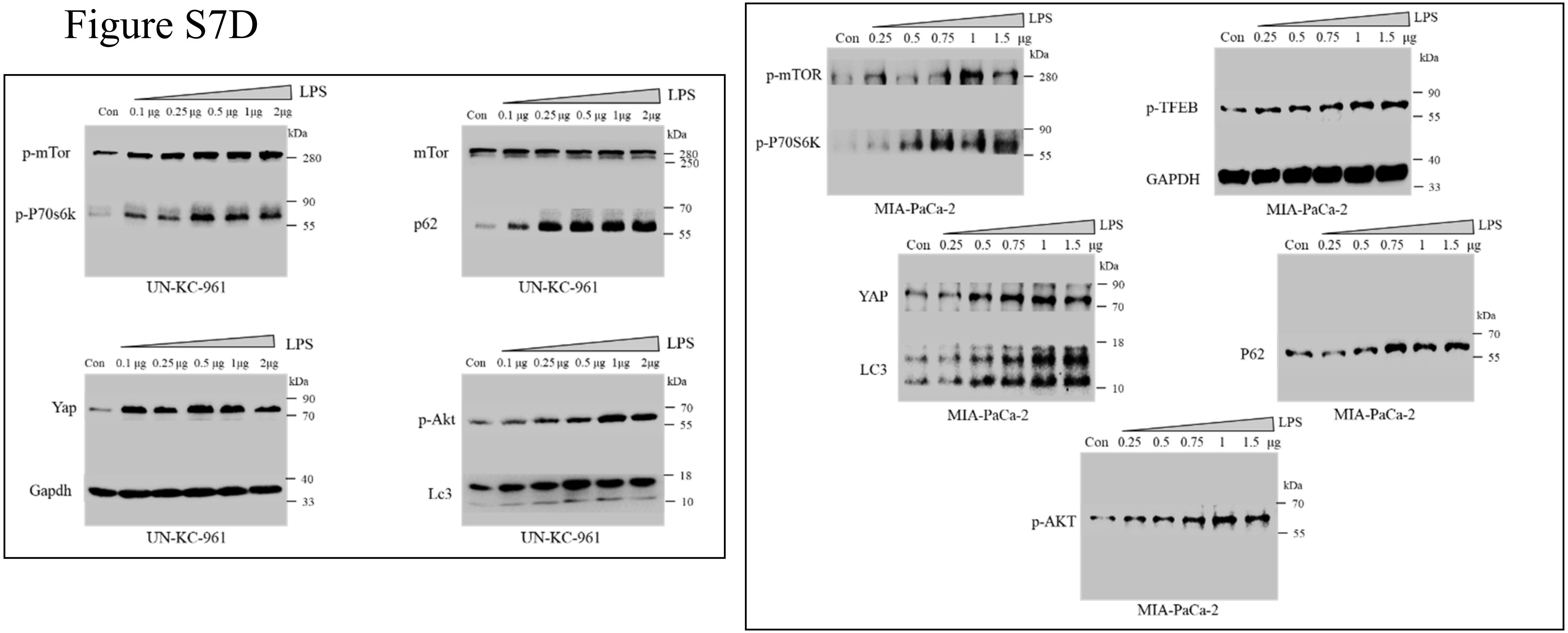

LPS
Con
0.1 µg
0.25 µg
0.5 µg
1µg
2µg
kDa
p-mTor
p-P70s6k
mTor
p62
Yap
Gapdh
p-Akt
Lc3
UN-KC-961
0.25
0.5
0.75
1
1.5
µg
p-mTOR
p-P70S6K
p-TFEB
GAPDH
YAP
LC3
P62
p-AKT
MIA-PaCa-2

Figure S7E

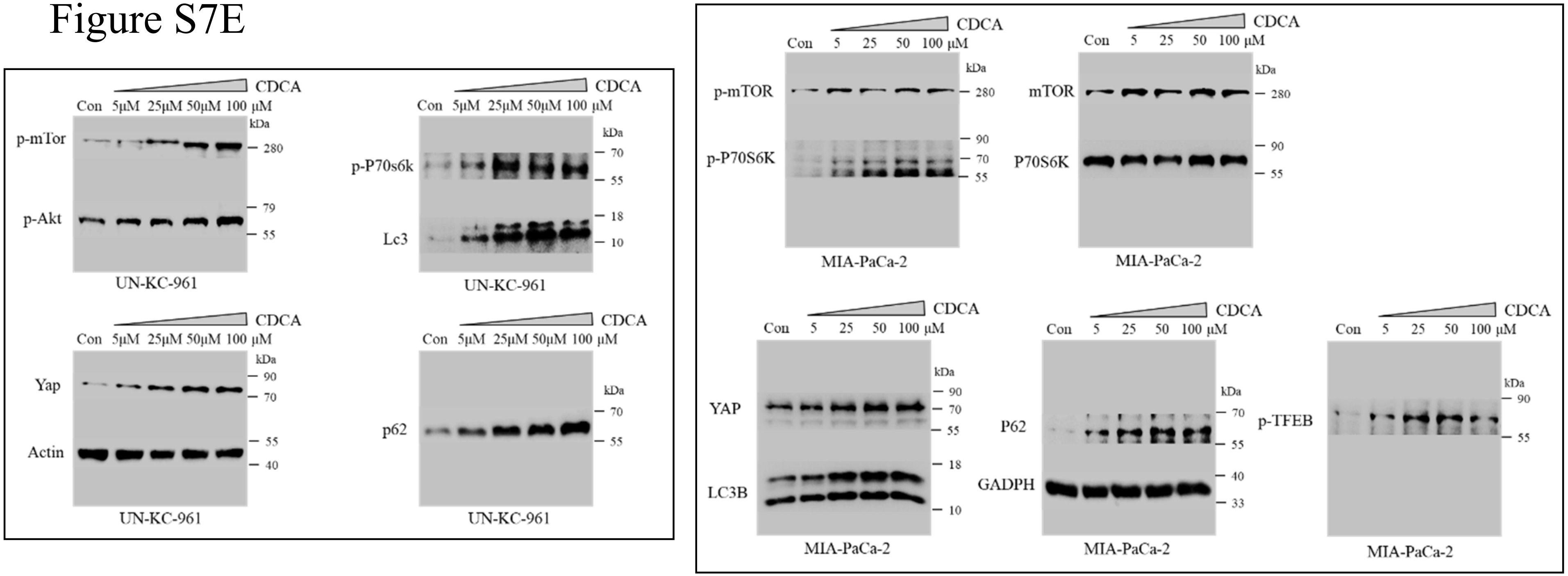
CDCA
Con 5μM 25μM 50μM 100 μM
kDa
p-mTor
280
p-Akt
79
55
UN-KC-961
p-P70s6k
70
55
Lc3
18
10
Yap
90
70
Actin
55
40
p62
MIA-PaCa-2
Con 5 25 50 100 μM
p-mTOR
mTOR
p-P70S6K
P70S6K
YAP
LC3B
P62
GADPH
p-TFEB
33

Figure S7F

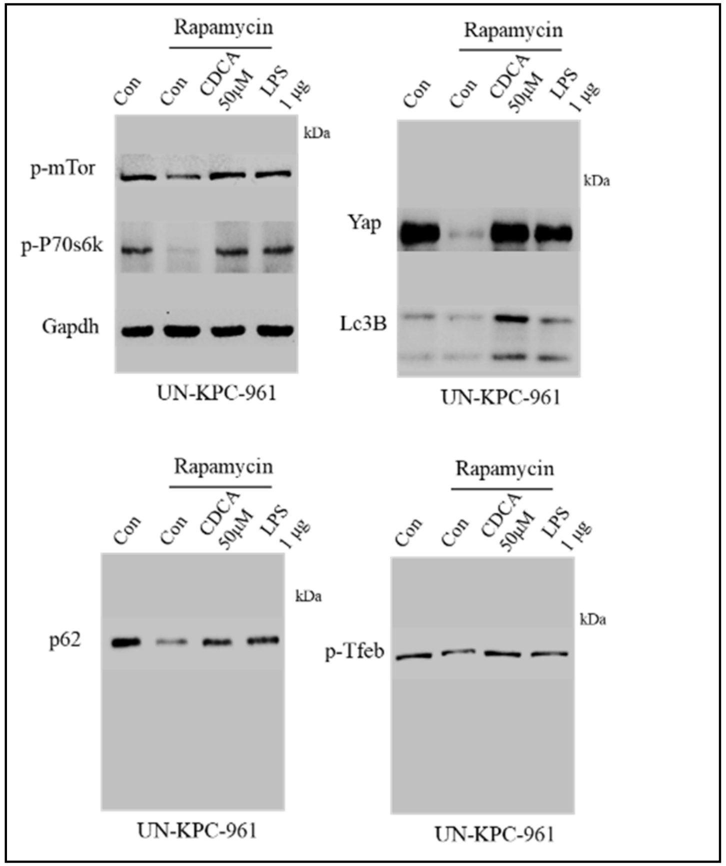
Rapamycin
Con
Con
CDCA 50μM
LPS 1 μg
kDa
p-mTor
p-P70s6k
Gapdh
UN-KPC-961
Yap
Lc3B
p62
p-Tfeb

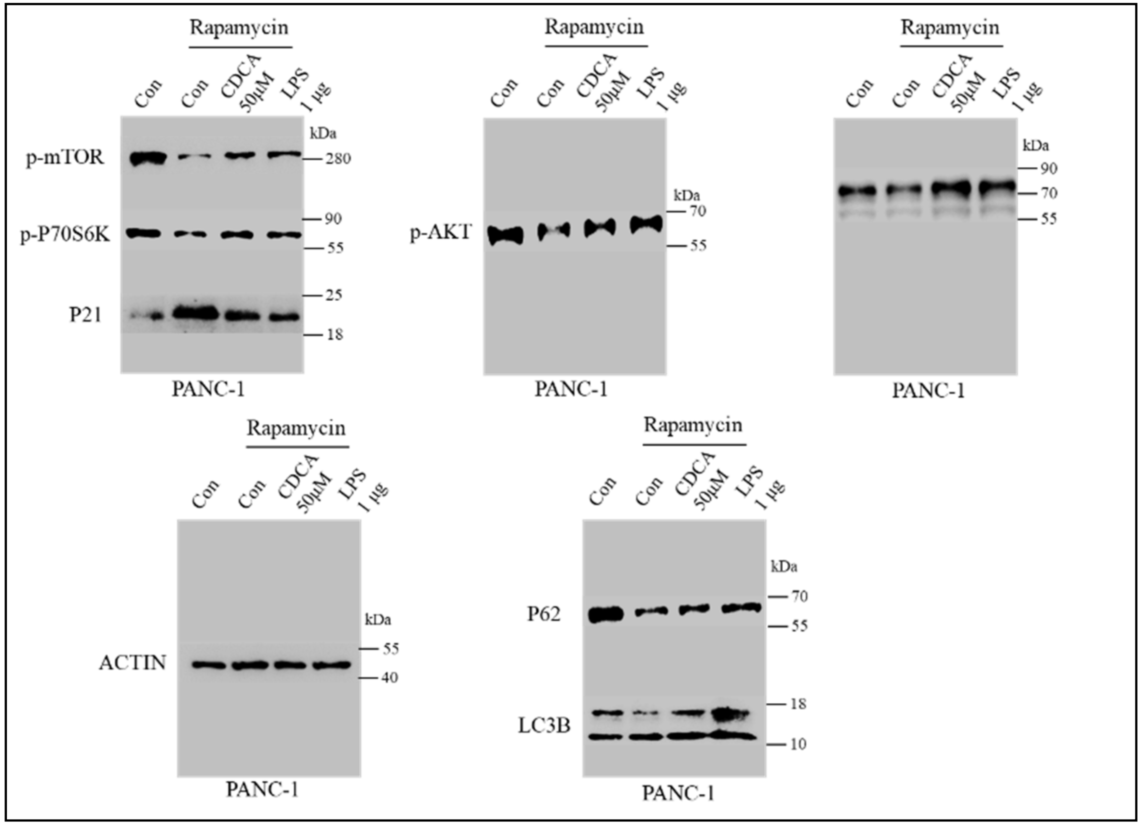
Rapamycin
Con
Con
CDCA 50μM
LPS 1 μg
kDa
p-mTOR
p-P70S6K
P21
280
90
55
25
18
PANC-1
p-AKT
70
ACTIN
40
P62
LC3B
10

Figure S8A

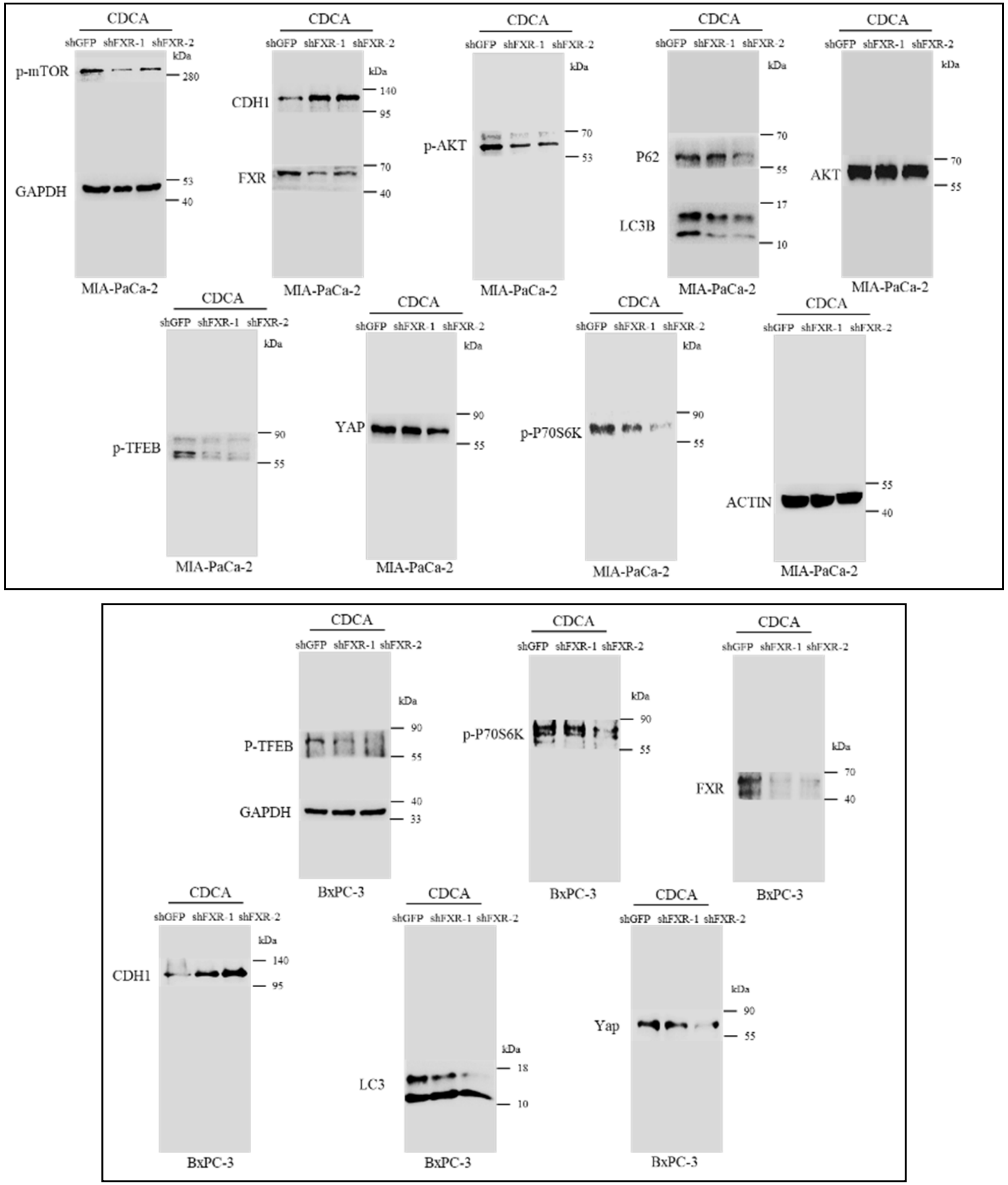

CDCA
shGFP shFXR-1 shFXR-2
kDa
p-mTOR
280
GAPDH
53
40
MIA-PaCa-2
CDH1
140
95
FXR
70
40
p-AKT
70
53
P62
70
55
LC3B
17
10
AKT
70
55
p-TFEB
90
55
YAP
90
55
p-P70S6K
90
55
ACTIN
55
40
P-TFEB
90
55
GAPDH
40
33
BxPC-3
p-P70S6K
FXR
70
40
CDH1
140
95
LC3
18
10
Yap
90
55

Figure S8B

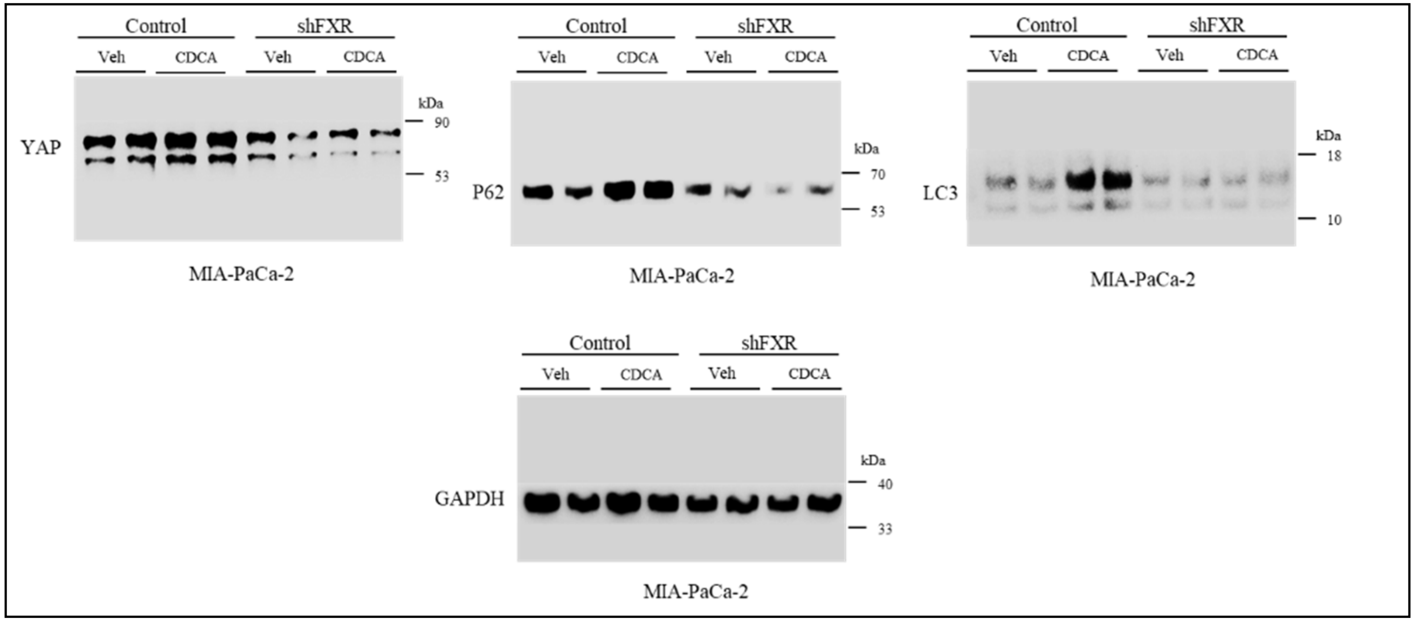

Control
shFXR
Veh
CDCA
Veh
CDCA
YAP
kDa
90
53
MIA-PaCa-2
Control
shFXR
Veh
CDCA
Veh
CDCA
P62
kDa
70
53
MIA-PaCa-2
Control
shFXR
Veh
CDCA
Veh
CDCA
LC3
kDa
18
10
MIA-PaCa-2
Control
shFXR
Veh
CDCA
Veh
CDCA
GAPDH
kDa
40
33
MIA-PaCa-2

Figure S8C

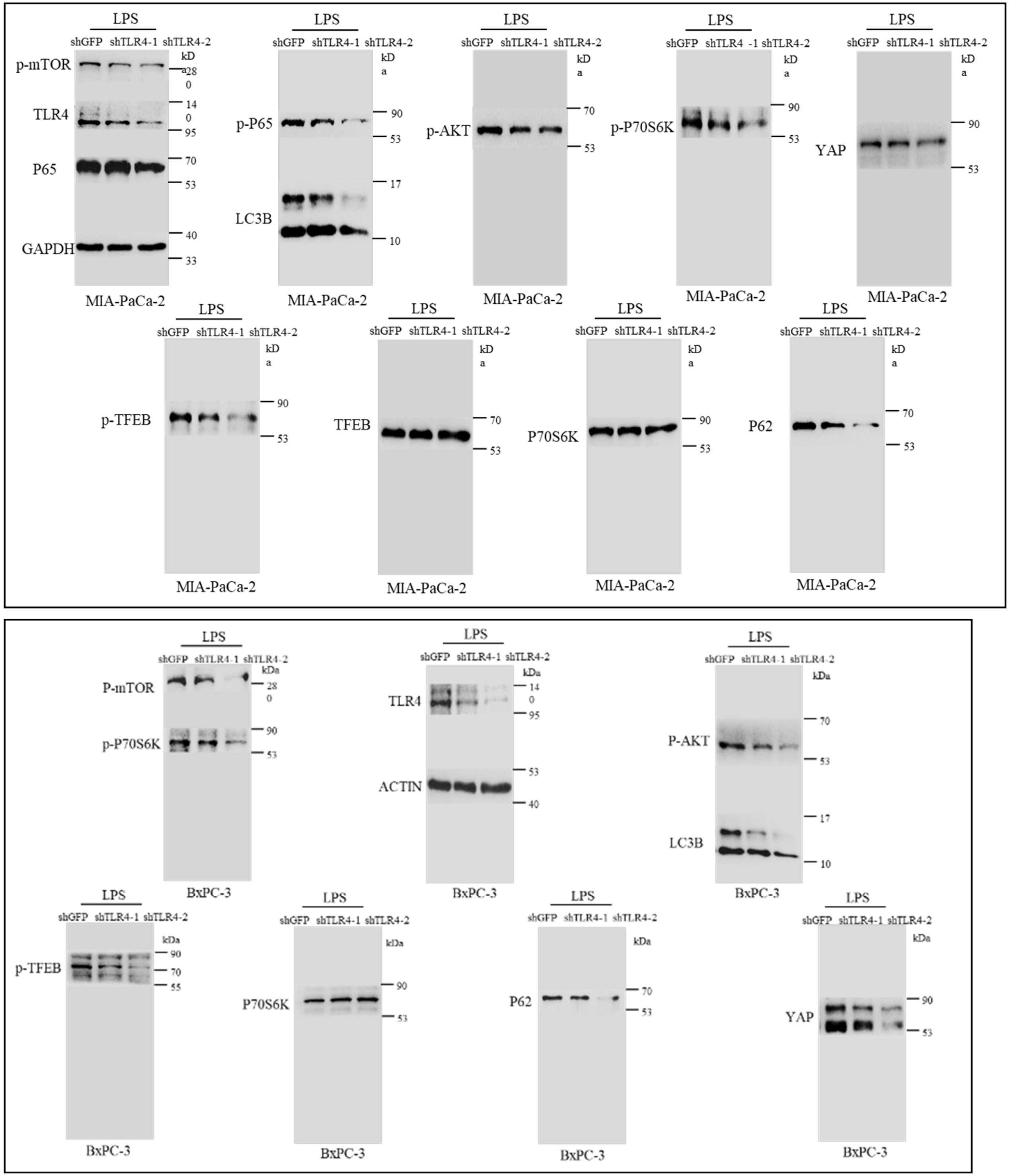
LPS
shGFP shTLR4-1 shTLR4-2
kDa
p-mTOR
TLR4
P65
GAPDH
p-P65
LC3B
p-AKT
p-P70S6K
YAP
p-TFEB
TFEB
P70S6K
P62
MIA-PaCa-2
P-mTOR
P-AKT
ACTIN
BxPC-3

Figure S8D

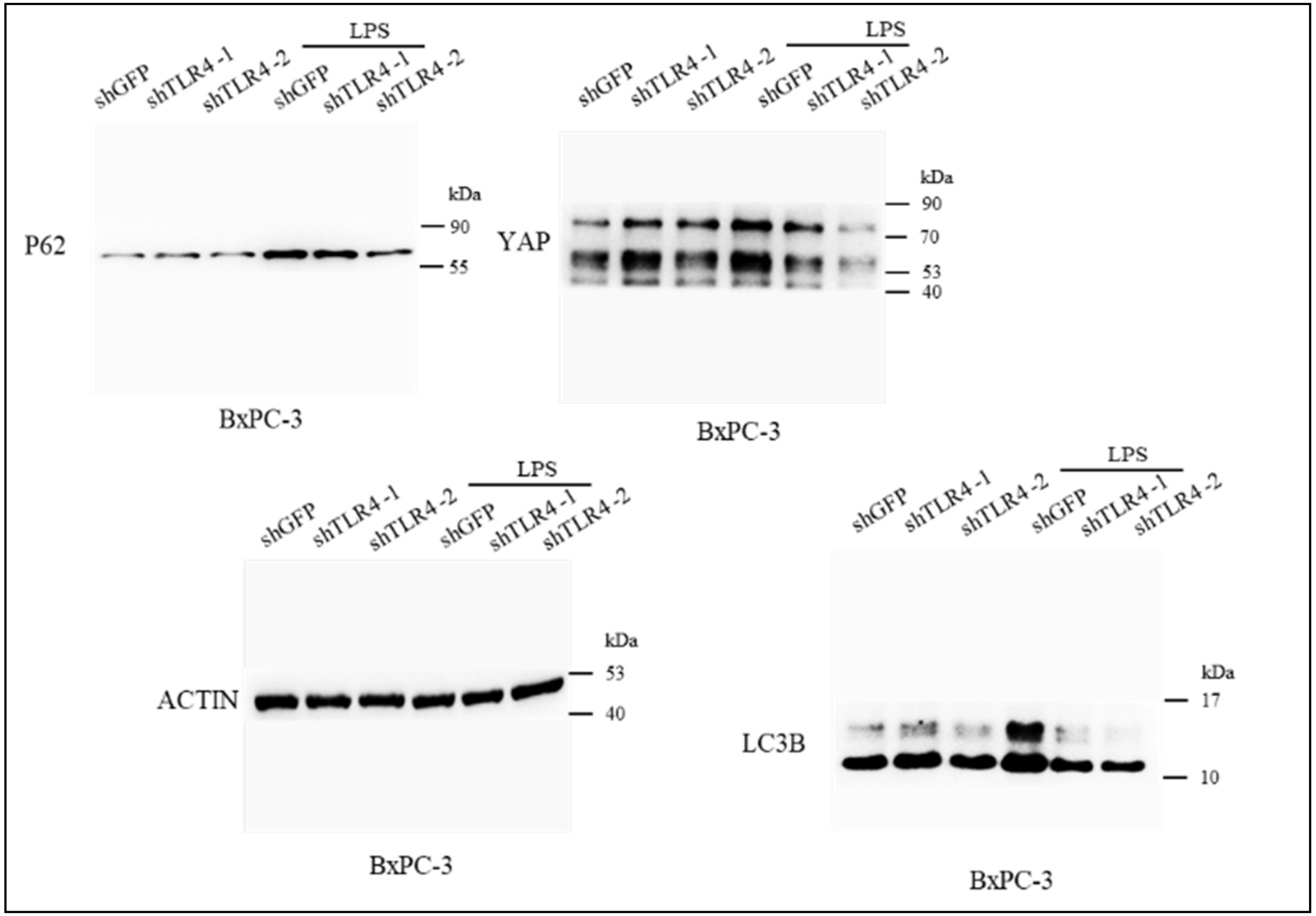
LPS
shGFP
shTLR4-1
shTLR4-2
shGFP
shTLR4-1
shTLR4-2
P62
kDa
90
55
BxPC-3
LPS
shGFP
shTLR4-1
shTLR4-2
shGFP
shTLR4-1
shTLR4-2
YAP
kDa
90
70
53
40
BxPC-3
LPS
shGFP
shTLR4-1
shTLR4-2
shGFP
shTLR4-1
shTLR4-2
ACTIN
kDa
53
40
BxPC-3
LPS
shGFP
shTLR4-1
shTLR4-2
shGFP
shTLR4-1
shTLR4-2
LC3B
kDa
17
10
BxPC-3

Supplementary Figure S3A

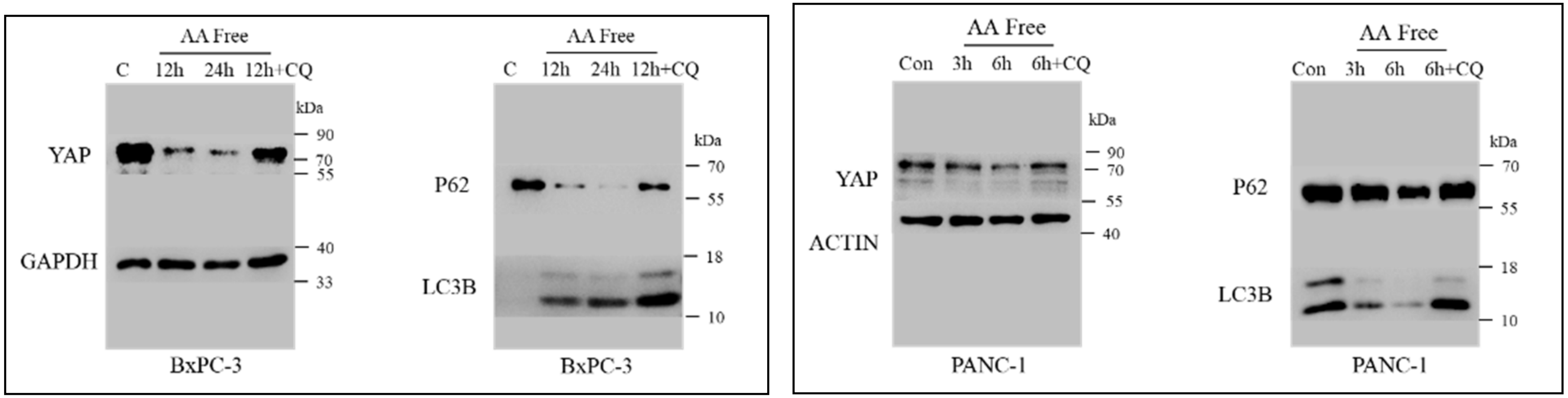

Supplementary Figure S3C

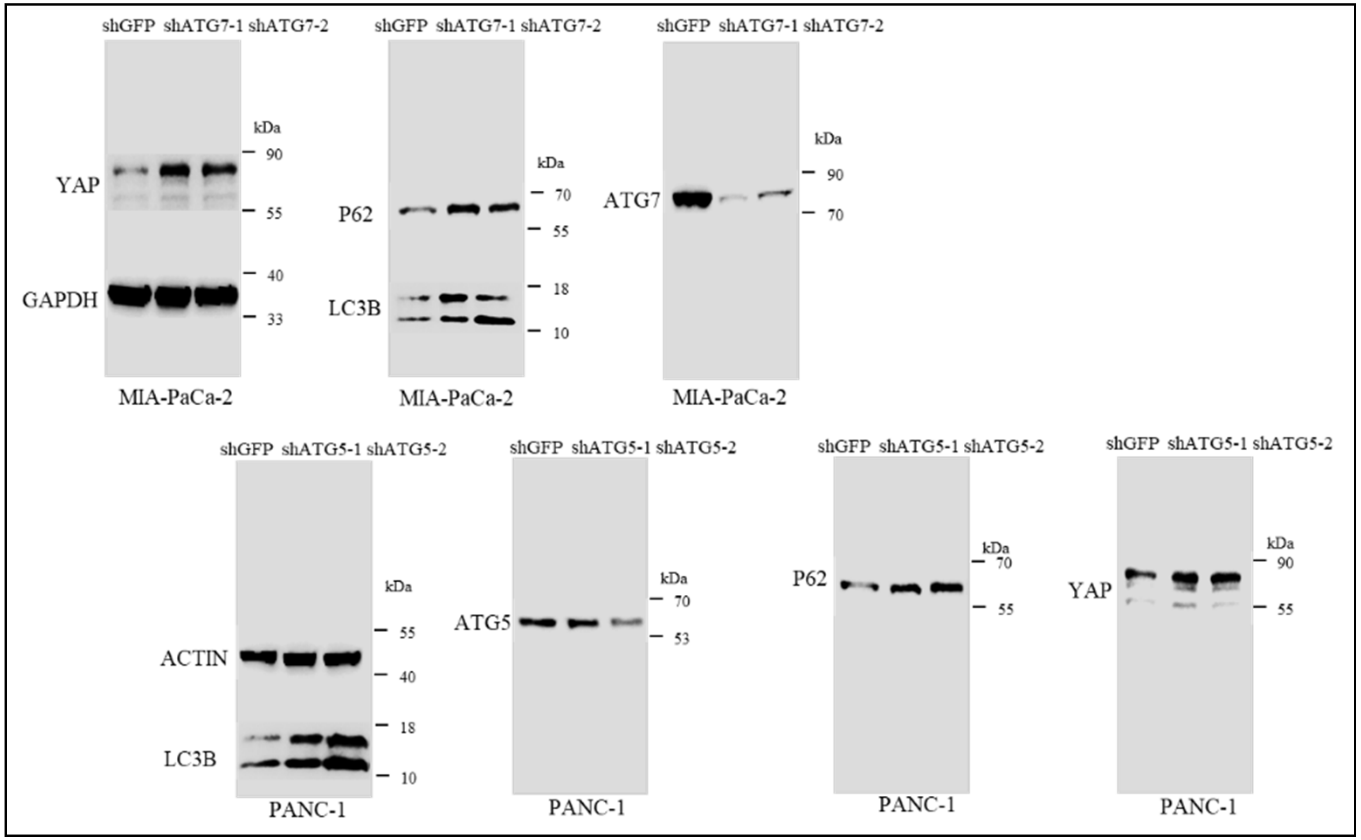

Supplementary Figure S6B

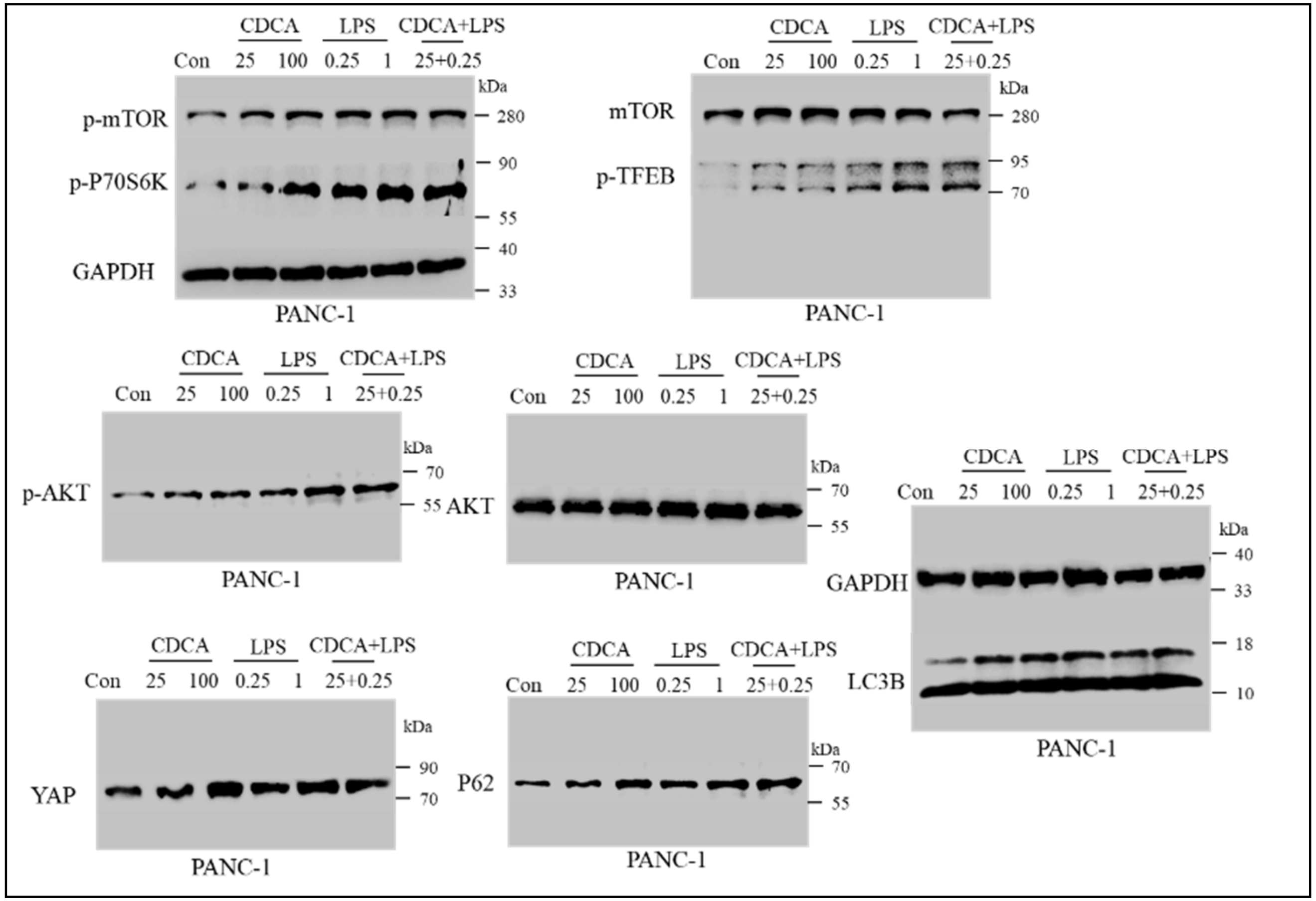

Supplement: Supplementary file 1 [file cancers-14-01407-s001.zip › cancers-1585928-original image.pdf]
