# Supplementary material for: Sequestration of Intestinal Acidic Toxins by Cationic Resin Attenuates Pancreatic Cancer Progression through Promoting Autophagic Flux for YAP Degradation
Source: Cancers (Basel). 2022 Mar 10;14(6):1407. doi: 10.3390/cancers14061407 (PMC8946475; doi:10.3390/cancers14061407)
Supplement: Supplementary file 1 [file cancers-14-01407-s001.zip › Supplementary file S1,Supplementary Figure 1 to 7.pdf]

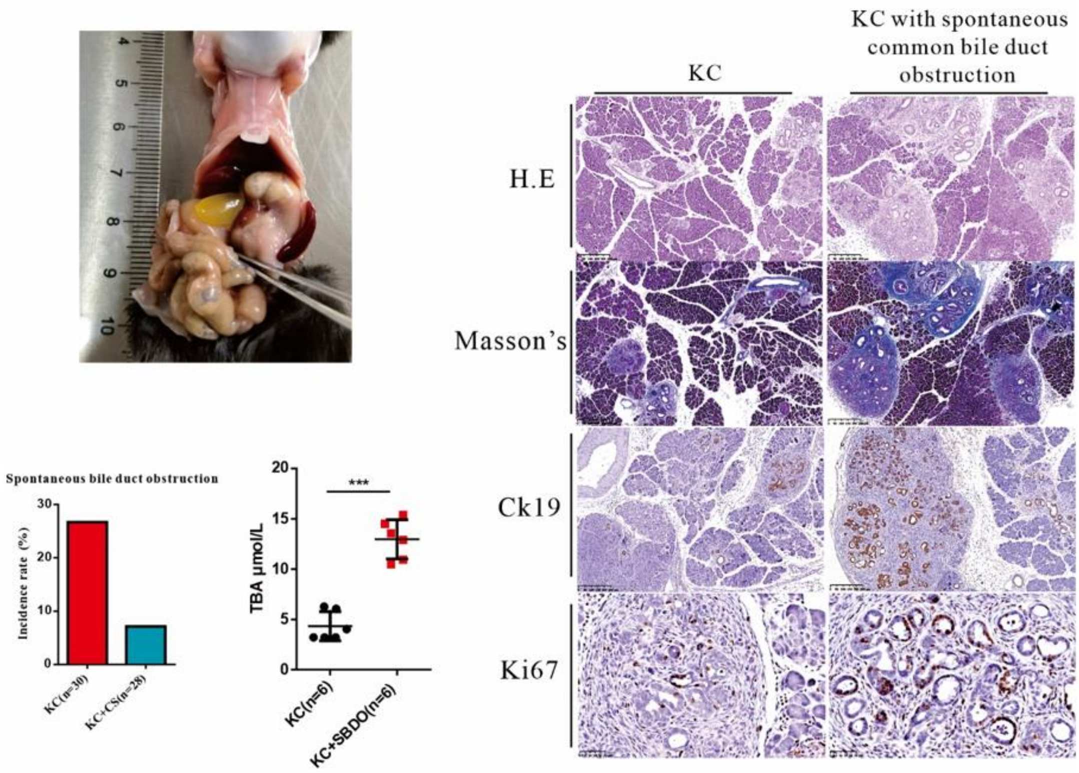

Supplementary Figure S1. KC mice spontaneously developed common bile duct obstruct. On the left side, a photo showed the spontaneous common bile duct obstruct and statistics for the incidence rate and serum TBA. On the right side, histologic and IHC analyses showed the pathologic differences between KC mice with or without spontaneous common bile duct obstruct. The scale bars for H&E, Masson's trichrome and Ck19 staining were 250μm while the scale bars for Ki67 staining were 50μm. Representative images of histology staining and IHC staining were shown. *P <0.05, **P < 0.01, ***P< 0.001.

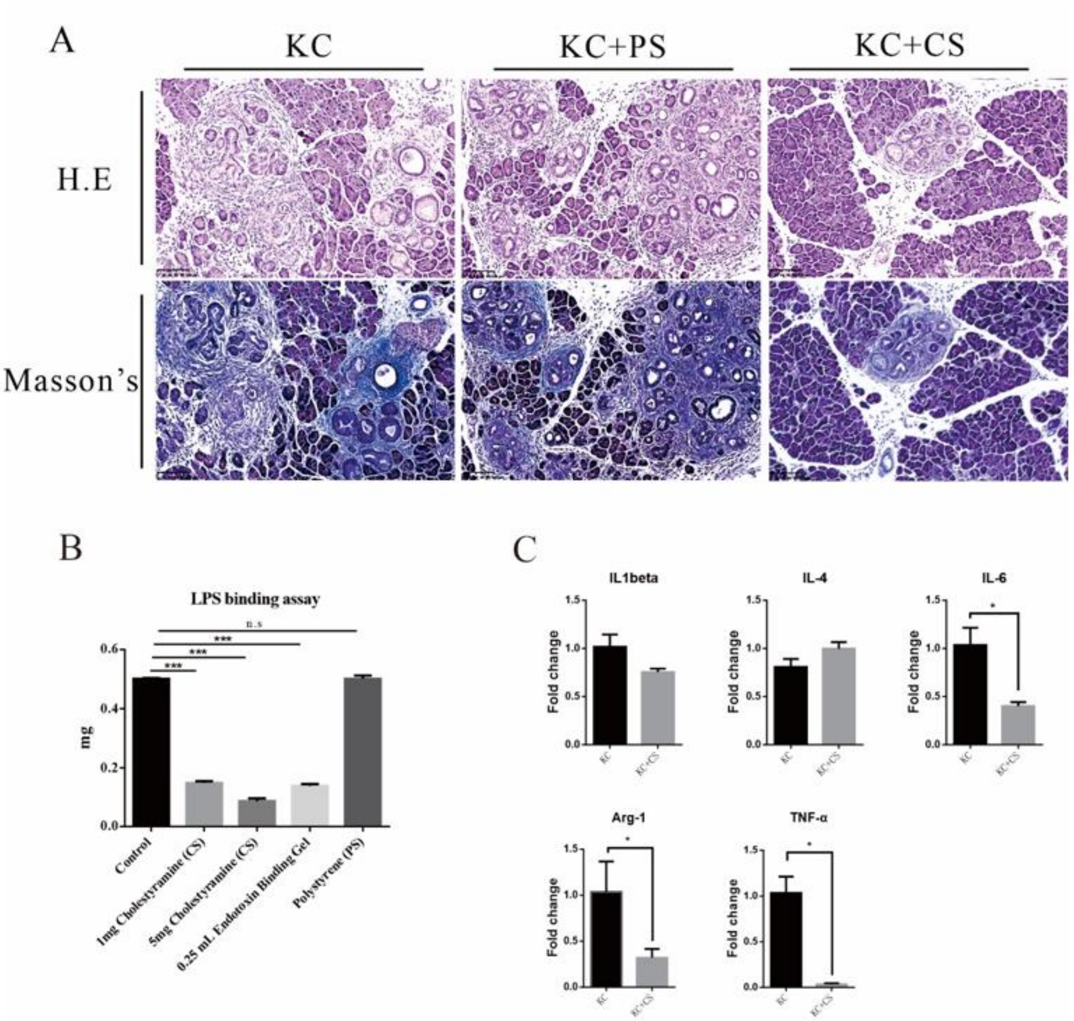

Supplementary Figure S2. A Histologic analysis (H&E and Masson's trichrome) of KC mice treated with vehicle, PS and CS. Scale bars =100μm. **B** The *in vitro* LPS binding assay. 0.5mg LPS was treated with different substrates for 30 mins and then measured the optical density at OD254 through spectrophotometer. **C** Comparation of inflammatory factor expressions between KC mice and KC mice treated with CS through q-PCR. Representative images of histology staining were shown. *P <0.05, **P < 0.01, ***P< 0.001.

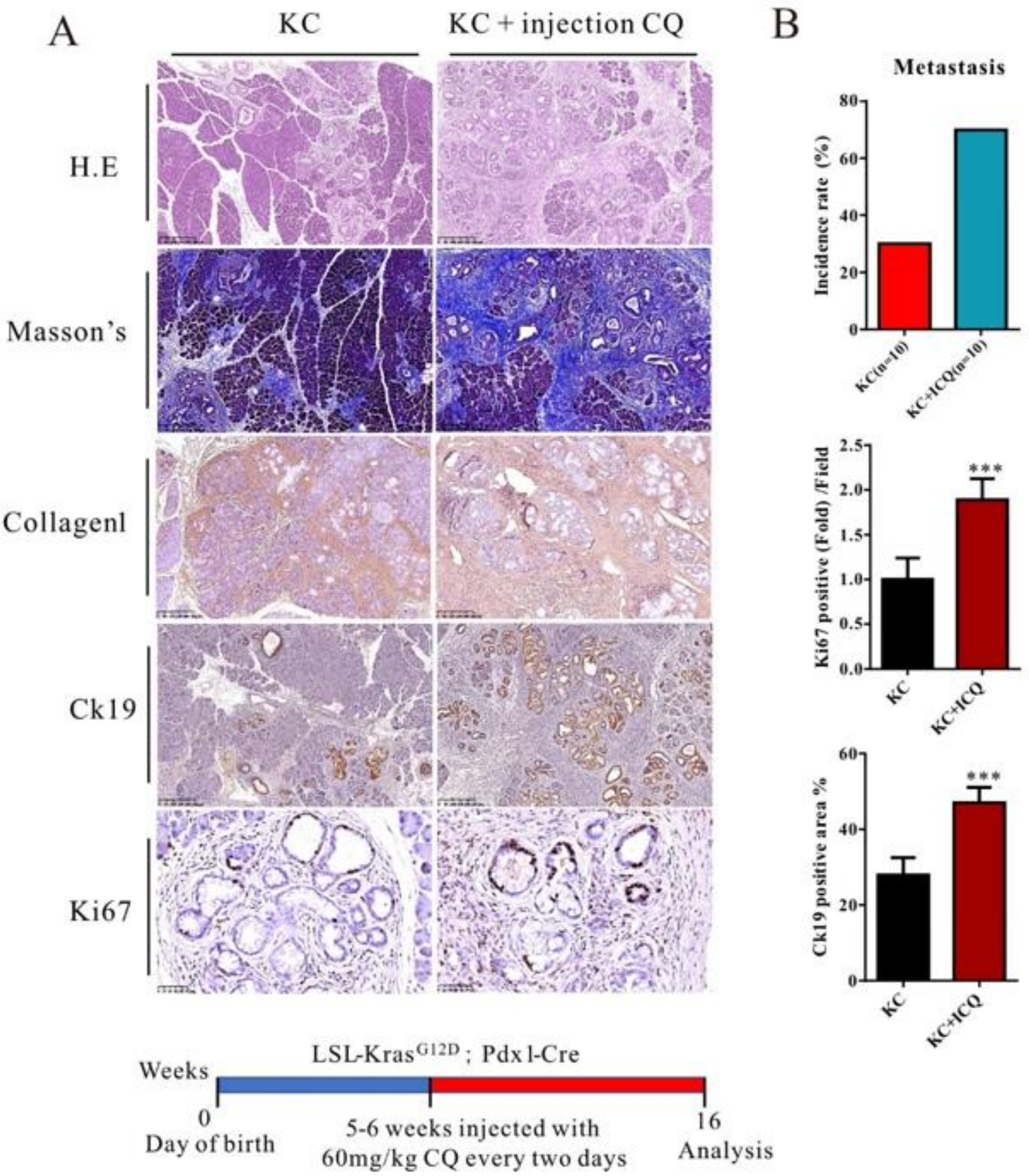

Supplementary Figure S3. A Western blot analyses of autophagic degradation of YAP in pancreatic cell lines under starving. **B** Cellular morphology of BxPC-3 under feeding and starving. The red circle indicates the cell junction. **C** Western blot analyses of autophagic degradation of YAP in pancreatic cell lines with ATG5 or ATG7 shRNA knockdown. Representative images of experiments repeated 2~3 times.

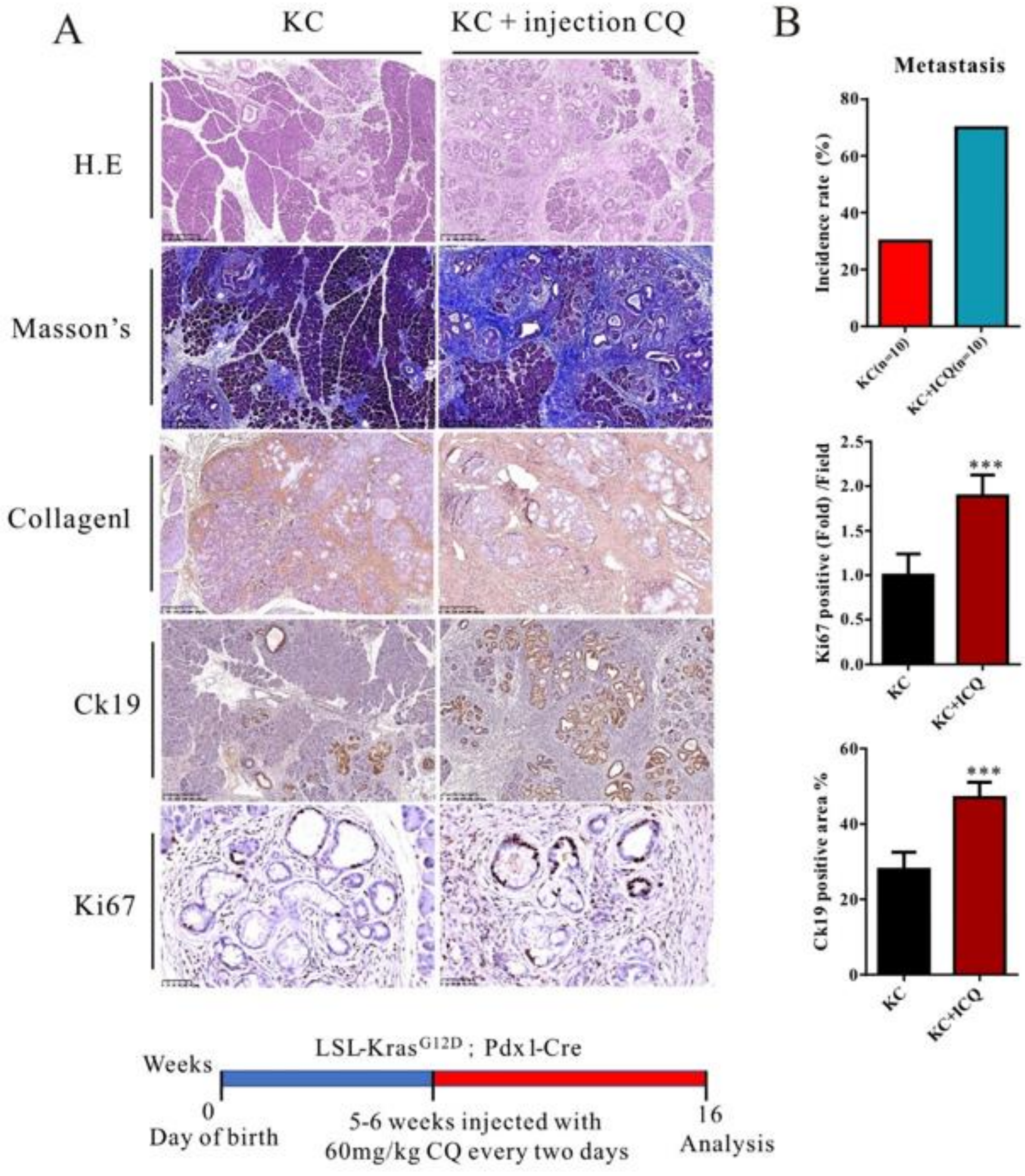

Supplementary Figure S4. A histologic and IHC analyses showed the pathologic differences between KC mice with or without 60mg/kg CQ injection. KC+vehicle (n=10) and KC+CQ injection (n=10). For H&E, Masson's trichrome, type 1 collagen and Ck19 staining, the scale bars were 250 μm. For Ki67 staining, the scale bars were 50μm. **B** These statistics for the metastasis rate, Ck19 positive area and Ki67 density. Representative images of histology staining and IHC staining were shown. *P <0.05, **P < 0.01, ***P< 0.001.

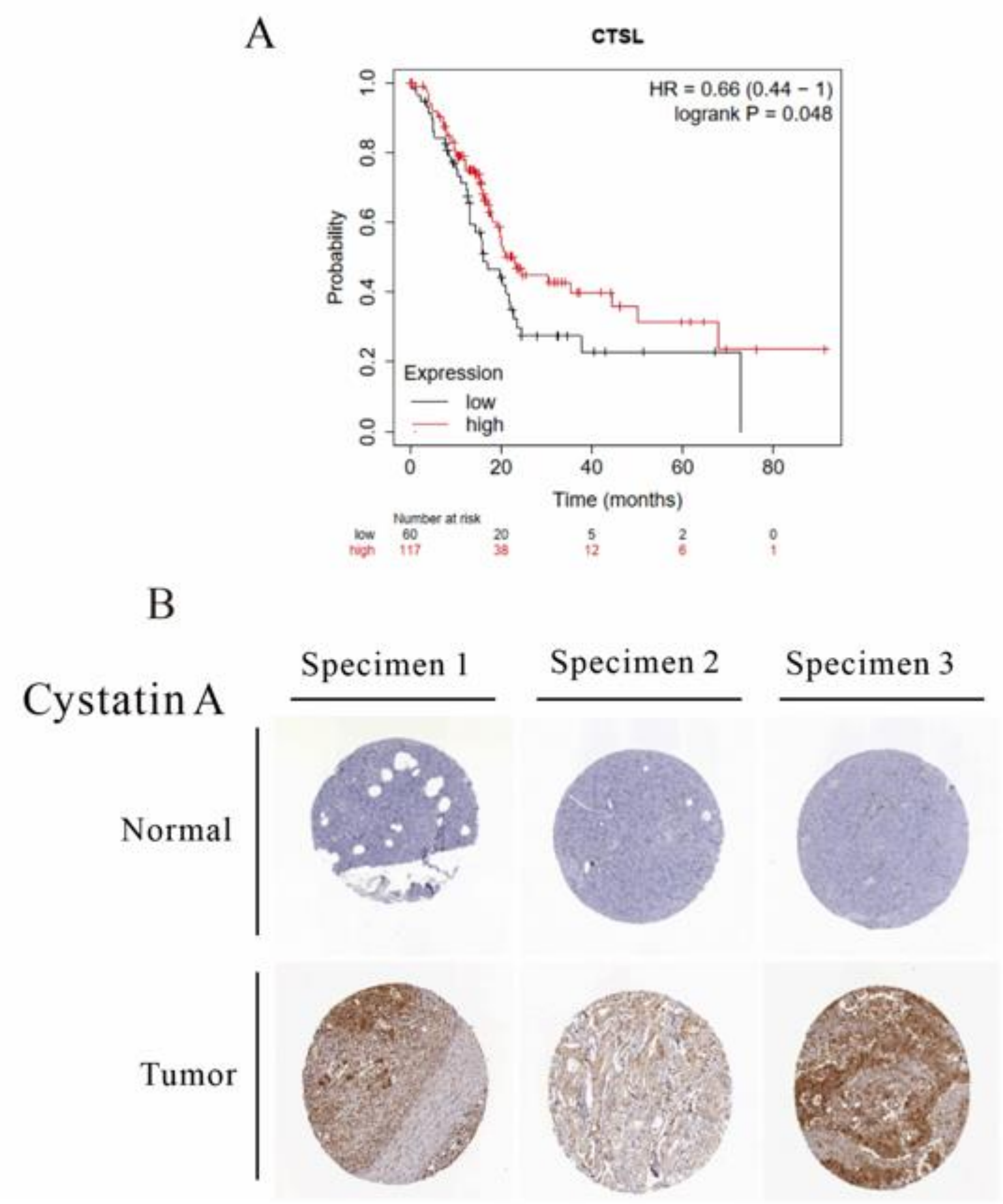

Supplementary Figure S5. A The Kaplan-Meier survival analysis of CTSL expression in pancreatic cancer based on Kaplan-Meier Plotter database (http://kmplot.com/analysis/). **B** Results of CSTA IHC staining of normal pancreas (not detected) and PDAC using HPA001031 antibody in the Human Protein Atlas database (https://www.proteinatlas.org/).

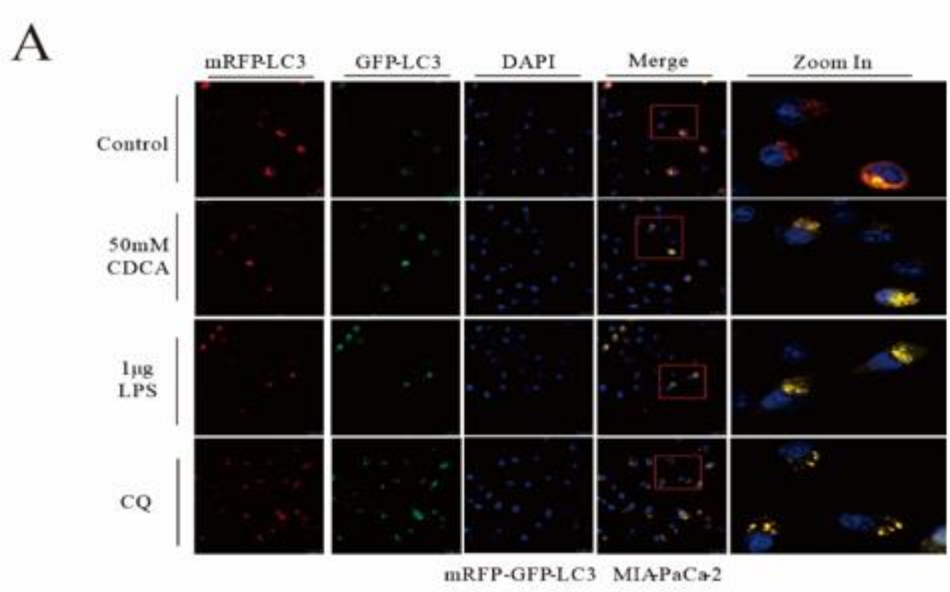

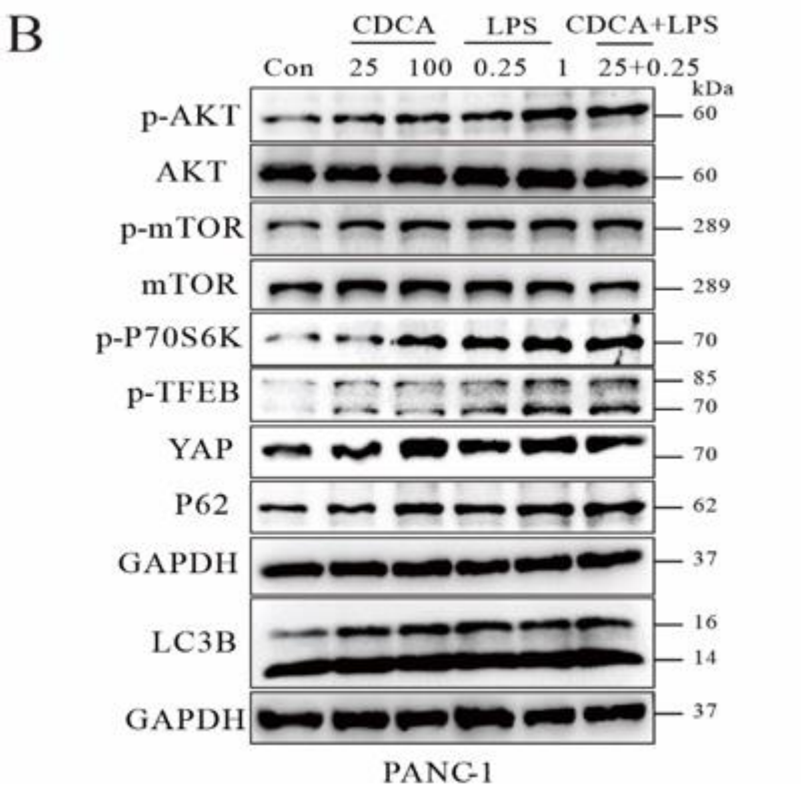

Supplementary Figure S6. A. a RFP-GFP-LC3 analyses for MIA-PaCa-2 cells treated with CDCA, LPS and CQ. **B** Western blot analysis for MIA-PaCa-2 cells treated with LPS (μg) or/and CDCA (μM) for 12h. Representative images of experiments repeated 2~3 times.

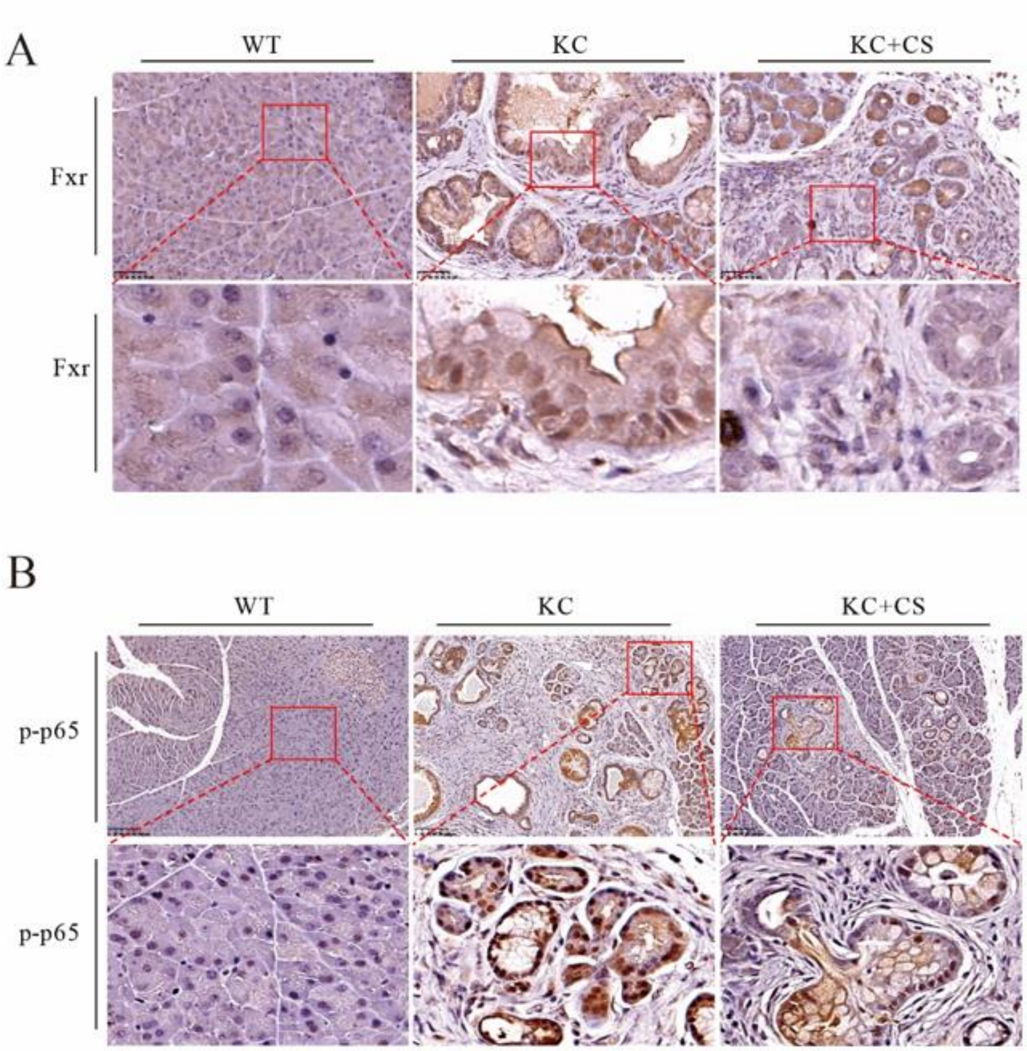

Supplementary Figure S7. A IHC staining for Fxr expressions in the pancreas WT, KC and KC plus CS mice. Scale bars = 50μm. **B** IHC staining for p-p65 expressions in the pancreas of WT, KC and KC plus CS mice. Scale bars = 100μm. Representative images of IHC staining were shown.
