# Supplementary material for: Sequestration of Intestinal Acidic Toxins by Cationic Resin Attenuates Pancreatic Cancer Progression through Promoting Autophagic Flux for YAP Degradation
Source: Cancers (Basel). 2022 Mar 10;14(6):1407. doi: 10.3390/cancers14061407 (PMC8946475; doi:10.3390/cancers14061407)
Supplement: Supplementary file 1 [file cancers-14-01407-s001.zip › Supplementary file S2,Details of reagents, plasmids.pdf]

Methods

Quantitative real-time RT-PCR

Quantitative real-time RT-PCR analysis of the expression of the inflammatory factors was performed using total RNA and the SYBR Green reagent (Bio-Rad) in CFX96 Real-Time System (Bio-Rad, Hercules, CA, USA). Gene expression levels were normalized to that of GAPDH. The results were analyzed using 2-ΔΔCt method. Primer sequence information used for qPCR analysis is listed below. The experiments were performed in triplicate and repeated twice.

In Vitro Sequestration of LPS by Cationic Resins

LPS was dissolved in pure water and specific adsorption was recorded at 258 nm. Cholestyramine, polystyrene powder, and Pierce High-Capacity Endotoxin Removal Resin (catalog no. 88270; Thermo Fisher Scientific) were mixed with LPS solution. The mixtures were incubated for 1 h. After centrifuging at 500g for 1 min, the supernatant was filtered through a 0.45-micron filter, and adsorption was measured at 258 nm for the unbound LPS. The experiment was repeated three times.

Reagents and antibodies

Reagents: Chloroquine diphosphate salt (Sigma, C6628); Lipopolysaccharides from Escherichia coli O111:B4 (Sigma, L2630); Chenodeoxycholic acid (Aladdin, C1049021); Rapamycin (MCE, HY-10219); MG132(MCE, HY-13259); ECL for Western Blot (Bio-Rad, #170-5061); H.E staining (Biosharp, BL700A); Masson's staining (Baso, BA4079A); Transwell (Falcon,353097); DAB for IHC (Origene, ZLI-9018); Paraformaldehyde (Sigma, 441244).

Primary Antibodies: YAP1 (CST, 14074); Type 1 Collagen-UNLB (Southern Biotech, 1310-61); P62 (Zenbio, 380612); CK19 (Abcam, ab52625); Ki67 (CST, 12202); Phospho-AKT (Ser473) (CST, 23430); AKT (CST, 9272); mTOR (CST, 2983); Phospho-mTOR (Ser2448) (CST, 5536); Phospho-Drosophila p70 S6 Kinase (Thr398) (CST,9209); p70 S6 Kinase (CST, 9092); Phospho-TFEB (Ser211) (Affinity, AF3708); TLR-4 (Santa Cruz Biotechnology, sc-293072); FXR (Proteintech, 25055-1-AP); LC3B (Abclonal, A19665); LC3 (Proteintech, 14600-1-AP); P21 for human (Zenbio, 382492); p21(Zenbio, 385235); Vimentin (Proteintech, 60330-1-Ig); ZO-1 (Santa Cruz Biotechnology, sc-8146); CDH1 (Abways, CY1155); Cyclin D1 (Abclonal, A19038); ATG7 (Abways, CY5658); ATG5 (Abways, CY5766); Occludin (Santa Cruz biotechnology, sc-8144); Cystatin A (Abmart, TD7458); Cathepsin L (Abcam, ab58991); HA-Tag (CST, 3724);Beta-ACTIN (Abclonal, AC026); GAPDH (Zenbio, 200306-7E4).

Secondary Antibodies: Anti HRP-linked-rabbit IgG (Santa Cruz Biotechnology, sc2313); Anti HRP-linked-mouse IgG (Santa Cruz Biotechnology, sc2314); Anti HRP-linked-goat IgG (Santa Cruz Biotechnology, sc2033);

Plasmids

Plasmid pLVX-Puro-CTSL-WT or pLVX-Puro-CTSL-C138S were stored in our laboratory. HA-CSTA and HA-CSTA-T96M were constructed into pLVX-Puro by double enzyme digestion and confirmed by sequencing analysis. CTSL-HA and CTSL-HA-C138S were previously constructed by Dr. Zhang Chunyan and stored at our lab. Tandem monomeric mRFP-GFP-tagged LC3 was a kind gift from Dr. Ying Tong. The shRNAs were cloned into pLKO.1. The sequence information of the primers used for variants and shRNA were as following.

Human-shCTSL-1: 5-AAGGCGATGCACAACAGATTA-3;
Human-shCTSL-2: 5-GAATTGCCTCAGCTACTCTAA -3;
Human-shATG5-1: 5-GATTCATGGAATTGAGCCAAT-3
Human-shATG5-2: 5-GCAGAACCATACTATTTGCTT-3
Human-shATG7-1: 5-GCCTGCTGAGGAGCTCTCCAT-3
Human-shATG7-2: 5-CCAGAGAGTTTACCTCTCATT-3
Human-shTLR-4-1: 5-CCAAGTAGTCTAGCTTTCTTA-3
Human-shTLR-4-2: 5-CGTTTGGTTCTGGGAGAATTT-3
Human-shFXR-1: 5-CCACTTCTTGATGTGCTACAA-3
Human-shFXR-2: 5-GCCTGACTGAATTACGGACAT-3
HA-HOMO-CSTA: Forward: CGCGGAATTCGCCACCATGTACCC ATACGACGTCCCAGAC TACGCTATACCTGGAGGCT
Reverse: GGCG GGATCC CTAAAAGCCCGTCA
HA-HOMO-CSTA-T96M: Forward: CGCGGAATTCGCCACCATGTACCC ATACGACGTCCC AGAC TACGCTATACCTGGAGGCT
Reverse: GGCG GGATCC CTAAAAGCCCATCAGC

Primers

Mouse-IL1-beta:
Forward: 5-GAAATGCCACCTTTTGACAGTG-3
Reverse: 5-TGGATGCTCTCATCAGGACAG-3
Mouse-IL4:
Forward: 5-GGTCTCAACCCCCAGCTAGT-3
Reverse: 5-GCCGATGATCTCTCTCAAGTGAT-3
Mouse-IL6:
Forward: 5-TAGTCCTTCCTACCCCAATTTCC-3
Reverse: 5- TTGGTCCTTAGCCACTCCTTC-3
Mouse-Arg1:
Forward: 5-CTCCAAGCCAAAGTCCTTAGAG-3
Reverse: 5-AGGAGCTGTCATTAGGGACATC-3
Mouse-TNF-alpha:
Forward: 5-CCCTCACACTCAGATCATCTTCT-3
Reverse: 5-GCTACGACGTGGGCTACAG-3
